# Corticosteroids inhibit *Mycobacterium tuberculosis*-induced necrotic host cell death by abrogating mitochondrial membrane permeability transition

**DOI:** 10.1038/s41467-019-08405-9

**Published:** 2019-02-08

**Authors:** Jessica Gräb, Isabelle Suárez, Edeltraud van Gumpel, Sandra Winter, Fynn Schreiber, Anna Esser, Christoph Hölscher, Melanie Fritsch, Marc Herb, Michael Schramm, Laurens Wachsmuth, Christian Pallasch, Manolis Pasparakis, Hamid Kashkar, Jan Rybniker

**Affiliations:** 1 Department I of Internal Medicine, University of Cologne, 50937 Cologne, Germany; 2 Center for Molecular Medicine Cologne (CMMC), University of Cologne, 50931 Cologne, Germany; 3 German Center for Infection Research (DZIF), Partner Site Bonn-Cologne, Cologne, Germany; 4 German Center for Infection Research (DZIF), Partner Site Hamburg – Lübeck – Borstel – Riems, 23845 Germany; 5 Excellence Cluster on Cellular Stress Responses in Aging-Associated Diseases (CECAD), University of Cologne, 50931 Cologne, Germany; 6 Institute for Medical Microbiology, Immunology and Hygiene (IMMIH), University of Cologne, 50935 Cologne, Germany; 7 Institute for Genetics, University of Cologne, 50674 Cologne, Germany

## Abstract

Corticosteroids are host-directed drugs with proven beneficial effect on survival of tuberculosis (TB) patients, but their precise mechanisms of action in this disease remain largely unknown. Here we show that corticosteroids such as dexamethasone inhibit necrotic cell death of cells infected with *Mycobacterium tuberculosis (Mtb)* by facilitating mitogen-activated protein kinase phosphatase 1 (MKP-1)-dependent dephosphorylation of p38 MAPK. Characterization of infected mixed lineage kinase domain-like (MLKL) and tumor necrosis factor receptor 1 (TNFR1) knockout cells show that the underlying mechanism is independent from TNFα-signaling and necroptosis. Our results link corticosteroid function and p38 MAPK inhibition to abrogation of necrotic cell death mediated by mitochondrial membrane permeability transition, and open new avenues for research on novel host-directed therapies (HDT).

## Abstract (web summary)

Corticosteroids are host-directed drugs that enhance survival of tuberculosis patients through unclear mechanisms. Here, Gräb et al. show that corticosteroids inhibit necrotic death of cells infected with *Mycobacterium tuberculosis* by facilitating MKP-1-dependent dephosphorylation of p38 MAPK.

## Introduction

M*ycobacterium tuberculosis* (*Mtb*), the causative agent of tuberculosis (TB), is the major killer among infectious agents which led to 1.7 million deaths in 2016 (ref. ^1^). Clinical management of TB consists of combinations of antibiotics for several months, a concept which becomes increasingly complicated in times of rising numbers of multi-drug resistant *Mtb*-isolates^1^. Adjunctive host-directed therapy (HDT) might improve and accelerate treatment by modifying host pathways targeted by the intracellular pathogen *Mtb*^2–6^. One possible approach is to inhibit cell death induced by phagocytosed *Mtb* which can prevent mycobacterial spread, tissue damage, and hyperinflammation^3^. Successful implementation of such drugs requires fundamental understanding of cell-death mechanisms exploited by *Mtb*. However, the molecular pathways involved in the manipulation of host cell death, and the consequence of this on the outcome of the infection, remain highly controversial^7–9^. Since lytic cell death seems to benefit the mycobacteria by enabling dissemination inside the human body and spread to other individuals, current research efforts focus on elucidating the exact mechanisms of *Mtb*-induced necrosis, which occurs once intracellular bacteria enter their replicative mode. Initially, in vitro studies as well as data derived from zebrafish infection models suggested that tumor necrosis factor alpha (TNFα) and mixed lineage kinase domain-like- (MLKL) driven necroptosis is the main cell-death pathway exploited by *Mtb*^10,11^. These findings were linked to clinical data showing hyperinflammation and increased mortality in Vietnamese patients with TB meningitis (TBM) carrying a specific leukotriene A4 hydrolase (LTA4H) promoter genotype leading to elevated TNFα levels^11,12^. However, this LTA4H genotype was not associated with increased severity and frequency of pulmonary TB in non-Asian patients^13^. A recent clinical study performed in Indonesia found no correlation between LTA4H promoter polymorphism and TBM outcome^14^. Another study showed that necroptotic signaling is primed in *Mtb-*infected macrophages, but its pathophysiological consequence in disease is restricted^15^. Therefore, while it is clear that host cell death significantly affects the outcome of TB, it is critically important to understand the precise role of TNFα signaling and necroptosis in the pathogenesis of *Mtb* to effectively interfere with these pathways by HDT.

Currently, corticosteroids, such as dexamethasone, represent the only clinically approved adjunctive chemotherapeutics for TB^3,16^. However, their exact mechanism of action is ill-defined despite proven beneficial effect on survival of TB patients^17^. It is assumed that corticosteroids function via systemic suppression of TNFα in hyper-inflammatory states of the disease^12^. A direct cytoprotective effect of corticosteroids, which are extensively used in multiple other inflammatory diseases, has not been described so far.

Exploiting a high-throughput chemical genetics approach selecting for small molecules that abrogate *Mtb*-induced death of human phagocytes, we identified several clinically applied corticosteroids that protected *Mtb*-infected cells as efficiently as antituberculous antibiotics.

This key finding here provides an excellent opportunity to gain fundamental insight into mechanisms of *Mtb*-induced host cell death and its impact on a clinically approved therapeutic intervention. Using corticosteroids as a starting point, we systematically investigated cellular machineries executing apoptosis, necroptosis, and alternative forms of regulated necrosis. Our mechanistic studies identified the mitochondrial permeability transition pore (mPTP) to be involved in necrotic cell death upon *Mtb* infection. The data obtained suggest that *Mtb* controls necrosis by manipulating mitochondrial membrane integrity and successful therapeutic interventions ultimately target mitochondria and interfere with TB pathogenesis.

## Results

### Corticosteroids potently inhibit *Mtb*-induced cell death

**Fig. 1:**
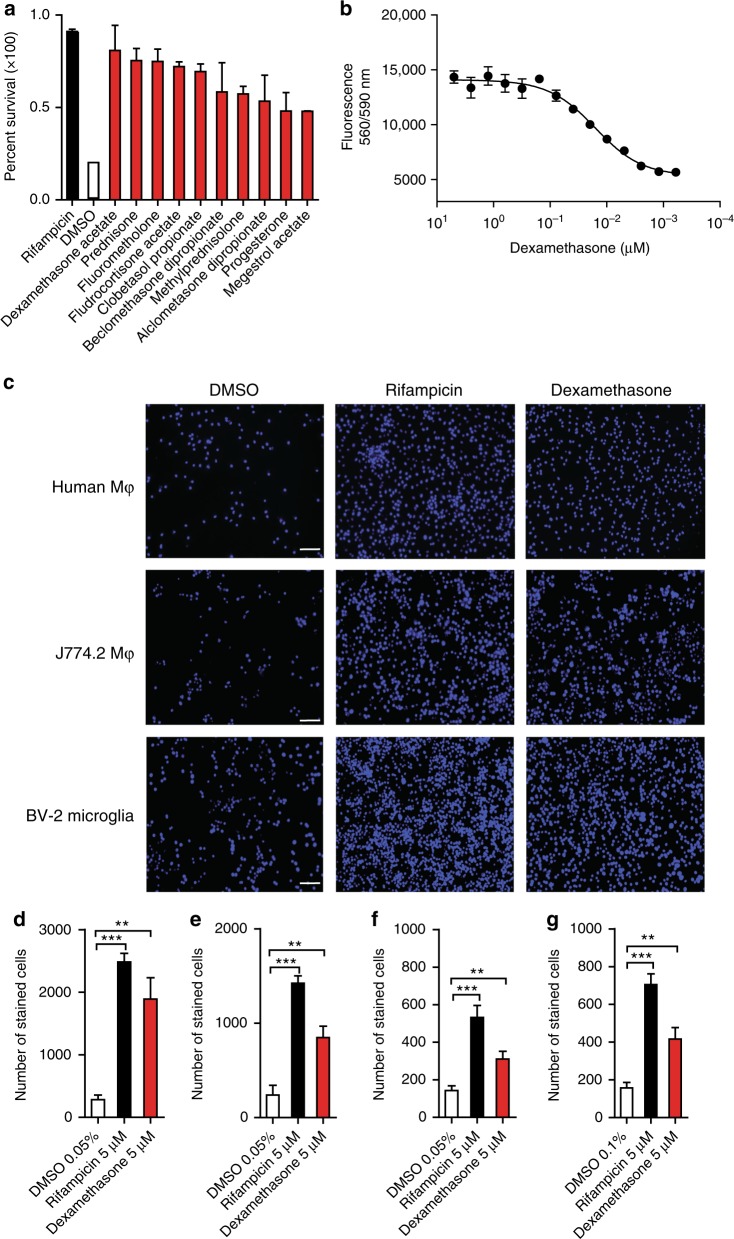
Corticosteroids abrogate cytotoxicity in *Mycobacterium tuberculosis (Mtb)*-infected cells. **a** Protective effect of dexamethasone and other corticosteroids (10 µM) in *Mtb-*infected MRC-5 lung fibroblasts (multiplicity of infection (MOI) 10). Host cell viability was quantified using PrestoBlue. Data derived from high-throughput screen with measurements of duplicate assay plates. **b** Dose–response curve of dexamethasone in *Mtb*-infected MRC-5 lung fibroblasts analyzed using the fibroblast survival assay (FSA). **c** Representative fluorescent microscopy images of *Mtb-*infected primary human macrophages (Mφ; MOI 1), J774.2 Mφ (MOI 5), and BV-2 microglia (MOI 5) treated with dexamethasone (5 µM), rifampicin (5 µM), or DMSO (0.05%). Nuclei were stained with 4′,6-diamidino-2-phenylindole (DAPI; scale bar: 100 µm). Images are representative of two to three individual experiments with multiple replicates. **d**–**g** Dexamethasone (5 µM)-treated J774.2 mouse Mφ (**d**), primary human Mφ from healthy donors (**e**) and TB patients (**f**), and BV-2 mouse microglia (**g**) were infected with the wild-type *Mtb* strain Erdman at varying MOI and surviving cells were stained with DAPI to determine the number of living cells 48 h post infection. Data from one experiment with duplicates are shown in **b**; data were pooled from two (**d**, **f**, **g**) or three (**e**) independent experiments with multiple replicates. Results are expressed as the mean ± SEM. Statistical analysis were performed by unpaired *t*-test (**p ≤ 0.01; ***p ≤ 0.001)

Co-incubation and infection of phagocytes with *Mtb* inevitably leads to host cell death which is primarily mediated by the mycobacterial ESX-1 type VII secretion system, an essential virulence factor^18^. Massively attenuated mycobacteria such as the Bacille Calmette-Guérin vaccine strain fail to kill host cells which makes *Mtb*-induced cell death or cell survival an attractive readout for host-directed drug screens^18^. When testing pharmacologically active and approved drugs in a high-throughput chemical genetic screen selecting for compounds that abrogate *Mtb*-dependent cell death^19,20^, we detected a series of corticosteroids as potent hit compounds that protected *Mtb*-infected MRC-5 human lung fibroblasts as efficiently as known anti-mycobacterial drugs (Fig. 1a; Supplementary Fig. 1; Supplementary Table 1). The corticosteroid dexamethasone was highly cytoprotective even at low nanomolar concentrations (IC_50_: 15 nM) (Fig. 1b). Cell death was also abrogated in *Mtb*-infected J774 mouse macrophages (Mφ) (Fig. 1c, d) and in human Mφ isolated from healthy donors and treatment naïve TB patients **(**Fig. 1c, e, f**)**. Since corticosteroids are extensively used as adjunctive therapy in TB affecting the central nervous system we also tested for survival of pre-treated and infected microglial cells. Chemical treatment with dexamethasone led to strong survival of BV-2 microglial cells comparable to other infected phagocytes (Fig. 1c, g).

### Dexamethasone abrogates p38 MAPK phosphorylation

**Fig. 2:**
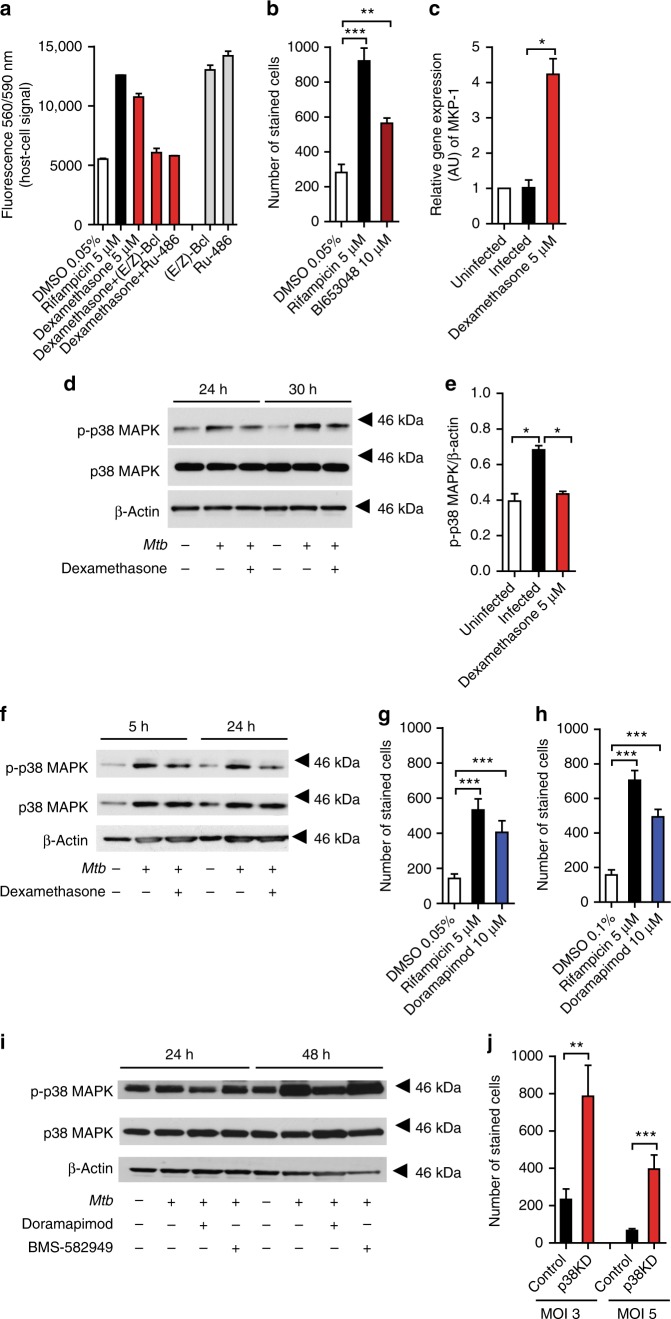
The protective effect of dexamethasone is mediated by MKP-1 and p38 MAPK inhibition. **a** Inhibition of MKP-1 by (E/Z)-Bcl hydrochloride or inhibition of the glucocorticoid receptor (GR) by Ru-486 in *Mtb-*infected MRC-5 lung fibroblasts (MOI 10) abrogates the protective effect of dexamethasone (5 µM) on host cell survival. Viable fibroblasts were quantified 72 h post infection using PrestoBlue. **b** Protective effect of the GR agonist BI653048 (10 µM) in *Mtb-*infected human Mφ (MOI 1). Cells were stained with DAPI and the number of living cells was determined 48 h post infection. **c** Dexamethasone (5 µM) treatment increases MKP-1 expression in infected MRC-5 lung fibroblasts 5 h after infection. Relative expression of MKP-1 was measured by quantitative real-time PCR (qRT-PCR). **d**–**f** Mtb induces p38 MAPK activation in J774.2 Mφ (**d**, **e**) and MRC-5 lung fibroblasts (**f**). Whole-cell lysates were obtained from infected cells treated with or without dexamethasone (5 µM) and equal amounts of protein were subjected to western blot analysis to determine the levels of phosphorylated and total p38 MAPK. β-Actin was used as a loading control. Images are representative of two to three individual experiments. **g**, **h** Cytoprotective effect of the p38 MAPK inhibitor doramapimod (10 µM) in *Mtb*-infected human Mφ from healthy donors (**g**) and TB patients (**h**). Cells were stained with DAPI and the number of living cells was determined 48 h post infection. **i** Doramapimod (10 µM) inhibits *Mtb*-induced p38 MAPK phosphorylation. Quantification of phosphorylated p38 MAPK in MRC-5 lung fibroblasts infected with *Mtb* and treated with the p38 MAPK inhibitors BMS-582949 (10 µM) or doramapimod by western blot. **j** Knock-down of p38 MAPK in *Mtb-*infected J774A.1 Mφ reduces mycobacterial cytotoxicity. Infected cells (MOI 3 and 5) were stained with DAPI and the number of surviving cells was determined after 48 h of infection. Data from one experiment with multiple replicates (**a**, **b**) or data pooled from four individual experiments (**c**) are shown; data from two individual experiments are shown in **e** and **h**; data from three independent experiments (**g**, **j**) with multiple replicates are shown as mean ± SEM. Analysis was done using unpaired *t-* test (*p ≤ 0.05; **p ≤ 0.01; ***p ≤ 0.001)

The corticosteroid-inducible protein mitogen-activated protein kinase (MAPK) phosphatase 1 (MKP-1; DUSP1) has emerged as a key molecule responsible for steroid-dependent effects on eukaryotic cells^21^. We speculated that inhibition of MKP-1 negatively affects the cytoprotective effects that were observed in dexamethasone-treated cells. Co-treatment of *Mtb*-infected human lung fibroblasts with dexamethasone and the specific MKP-1 inhibitor (E/Z)-BCI fully abrogated the dexamethasone protective effect (Fig. 2a). The substance alone showed no growth inhibitory effect on host cells (Fig. 2a). Similar observations were made when cells were co-treated with dexamethasone and the glucocorticoid receptor (GR) inhibitor Ru-486 or by single-agent treatment with the selective non-steroidal GR agonist BI653048 indicating that dexamethasone protects the cells via GR activation and MKP-1 upregulation (Fig. 2a, b; Supplementary Fig. 2). Strong MKP-1 upregulation of dexamethasone-treated *Mtb*-infected cells was confirmed by quantitative reverse transcriptase polymerase chain reaction (qRT-PCR) (Fig. 2c). MKP-1 specifically inactivates p38 MAPK, c-Jun N-terminal kinases (JNK), and extracellular signal-regulated kinases (ERK)^22^. We tested for phosphorylation of these kinases upon infection with *Mtb* and found p38 MAPK phosphorylation at several time points after infection of human lung fibroblasts and J774 Mφ (Fig. 2d–f). Dexamethasone treatment of infected cells inhibited p38 MAPK phosphorylation in both cell types (Fig. 2d–f; Supplementary Fig. 3). JNK or ERK phosphorylation was not observed at 5 and 24 h post infection (Supplementary Fig. 4a and b).

### Phosphorylation of p38 MAPK initiates cell death

Having identified p38 MAPK as a putative target of corticosteroids, we tested a series of p38 MAPK inhibitors and examined the survival of *Mtb*-infected cells. The data obtained showed that pamapimod, BMS-582949 and SB203580 did not interfere with *Mtb*-induced cell death at 10 µM (Supplementary Fig. 5a). In contrast, the highly potent and selective p38 MAPK inhibitor doramapimod was as protective as dexamethasone in several infected cell types including human Mφ isolated from healthy donors and treatment naïve TB patients (Fig. 2g, h; Supplementary Fig. 5b–e). To better understand these conflicting results we quantified p38 MAPK phosphorylation in BMS-582949 and doramapimod-treated MRC-5 lung fibroblasts after infection with *Mtb*. Interestingly, only doramapimod was capable of suppressing p38 MAPK phosphorylation in a sustainable manner whereas BMS-582949 failed to inhibit phosphorylation at 24 and 48 h post infection (Fig. 2i). This indicates that *Mtb* infection represents a potent inflammatory stimulus that can overcome chemical p38 MAPK inhibition in some of these substances. The underlying molecular mechanism however remains elusive.

To definitely clarify the role of p38 MAPK in *Mtb*-induced cell death, the expression of p38 MAPK was down regulated (p38KD) in J774 Mφ using selective siRNAs (Supplementary Fig. 6). Down-regulation of p38 expression significantly increased the survival of infected J774 Mφ, clearly indicating that p38 MAPK is involved in *Mtb*-induced cell death **(**Fig. 2j).

### Apoptosis is not the main mechanism for p38 MAPK mediated cell death

**Fig. 3:**
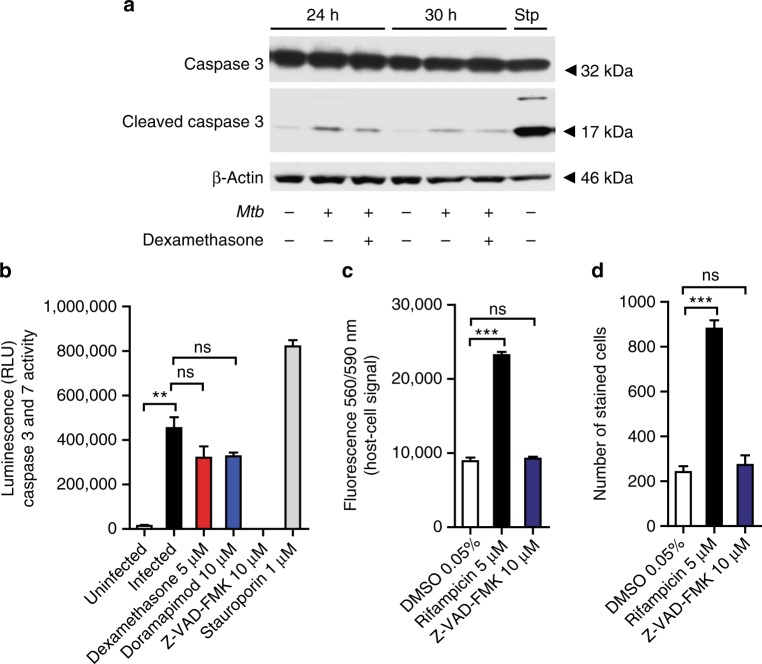
*Mtb*-induced cell death is independent of caspase activation. **a** Western blot analysis of proteolytic cleavage of caspase 3 in *Mtb*-infected J774.2 Mφ (MOI 5). Equal amounts of protein from infected J774.2 Mφ treated with or without dexamethasone (5 µM) were subjected to immunoblot analysis for cleaved caspase 3 following 24 and 30 h of infection. β-Actin was used as a loading control and staurosporine (1 µM) as a positive control. Images are representative of two individual experiments. **b** MRC-5 lung fibroblasts were infected with *Mtb* (MOI 10) and treated with dexamethasone (5 µM), doramapimod (10 µM), or Z-VAD-FMK (10 µM). After 48 h caspase 3 and caspase 7 activity was assessed using a luminescent probe. Uninfected and staurosporine (1 µM)-treated cells were used as controls. **c,** **d** Effect of caspase 3 and caspase 7 inhibition using the pan-caspase inhibitor Z-VAD-FMK (10 µM) on survival of infected MRC-5 lung fibroblasts (**c**) and human Mφ from healthy donors (**d**). Viable fibroblasts were detected using Prestoblue and Mφ were quantified by DAPI staining. Representative data from two experiments with multiple replicates are shown in **b**–**d**. Results are expressed as mean ± SEM. Analysis was done using unpaired *t*-test (ns not significant; **p ≤ 0.01; ***p ≤ 0.001)

To better understand the events following p38 MAPK phosphorylation as a consequence of *Mtb* infection, we carefully dissected several cell death pathways. MAP-kinases have been frequently associated with apoptotic processes such as the activation of pro-apoptotic Bcl-2 family proteins leading to caspase activation^7^. We first determined activation of executioner caspases in our infection models using immunoblots targeting cleaved caspase 3. *Mtb* infection led to some proteolytic activation of caspase 3 in J774 Mφ which was partially inhibited by dexamethasone and doramapimod (Fig. 3a). In a more sensitive luminescence-based caspase activity assay for both caspase 3 and caspase 7, a stronger activation signal was detected upon infection of human lung fibroblasts (Fig. 3b). p38 MAPK inhibition via dexamethasone or doramapimod led to a slight decrease in luminescent signal (Fig. 3b). We then treated cells with the pan-caspase inhibitor Z-VAD-FMK (*N*-benzlyoxycarbonyl-valyl-alanyl-aspartyl-fluoromethylketone). This substance led to full inhibition of caspase 3- and caspase 7 activation in infected cells (Fig. 3b). Assuming that p38 MAPK signaling leads to apoptosis execution via caspase 3/7, Z-VAD-FMK should be as cytoprotective as dexamethasone or doramapimod. However, caspase inhibition by Z-VAD-FMK failed to protect human lung fibroblasts and human primary Mφ from *Mtb*-induced cell death (Fig. 3c, d) excluding apoptosis as a responsible killing mechanism upon *Mtb* infection and indicating that caspase 3 activation is an incidental event of cell stress.

### p38 MAPK inhibition blocks secretion of necrosis markers

**Fig. 4:**
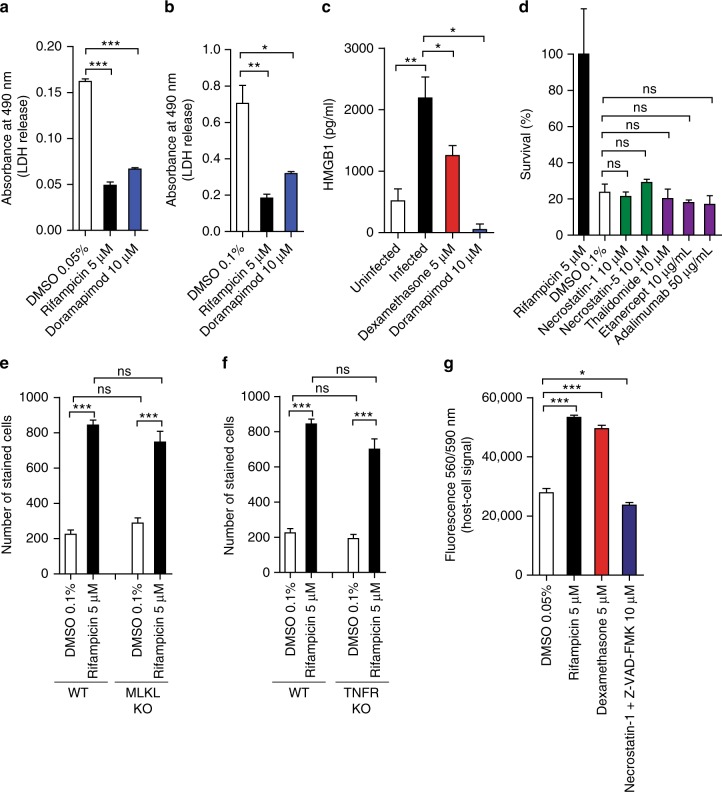
*Mtb* induces RIPK1 independent necrotic host cell death. **a**, **b** *Mtb*-infected MRC-5 lung fibroblasts were treated with doramapimod (10 µM). After 72 h lactate dehydrogenase (LDH) was quantified in the culture medium of infected cells (**a**). *Mtb*-infected human Mφ derived from TB patients were treated with doramapimod and LDH release was measured 48 h post infection in the supernatant (**b**). Data are representative of two individual experiments. **c** Dexamethasone (5 µM) and doramapimod (10 µM) reduce the release of the necrosis marker High-Mobility Group Protein B1 (HMGB1) in *Mtb*-infected MRC-5 lung fibroblasts. **d** Protective effect of necrostatin (Nec; 10 µM), etanercept (10 µg/ml), adalimumab (50 µg/ml), and thalidomide (10 µM) in *Mtb*-infected human Mφ from healthy donors. Cell viability was quantified by DAPI staining 48 h after infection. **e** Bone-marrow-derived Mφ (BMDM) from WT and mixed lineage kinase domain-like pseudokinase (MLKL)^−/−^ mice were infected with *Mtb* (MOI 10) and cell survival was assessed 48 h after infection using DAPI staining. **f** BMDM from WT and tumor necrosis factor receptor (TNFR)^−/−^ mice was infected with *Mtb* (MOI 10). Cell survival was determined 48 h after infection using DAPI staining. **g** MRC-5 lung fibroblasts were treated with Z-VAD-FMK (10 µM) and necrostatin-1 (10 µM) simultaneously and viable cells were analyzed 72 h post infection with TB using PrestoBlue. Representative data from at least two experiments with multiple replicates are shown in **a**, **b**, **d**–**f**. And data pooled from eight independent experiments are shown in **c**. Representative data from two experiments with multiple replicates are shown in **g**. Results are expressed as mean ± SEM. Analysis was done using unpaired *t*-test (ns not significant; *p ≤ 0.05; **p ≤ 0.01; ***p ≤ 0.001)

There is growing evidence that necrotic cell death plays a key role in pathogen-driven inflammation, cell to cell spread, and dissemination to new hosts^23^. We found large amounts of lactate dehydrogenase (LDH), a necrosis indicator, in the supernatant of *Mtb*-infected lung fibroblasts as well as in human Mφ isolated from healthy donors and treatment-naïve TB patient (Fig. 4a, b). In addition, the necrosis marker high-mobility group protein B1 (HMGB1) was released from *Mtb*-infected fibroblasts (Fig. 4c**)**. LDH and HMGB1 release was blocked in dexamethasone- or doramapimod-treated cells (lung fibroblasts, Mφ isolated from TB patients) (Fig. 4a–c). Collectively, these data link p38 MAPK activity to necrosis rather than apoptosis.

### TNFα and necroptosis are dispensable for corticosteroid function

The prototype of regulated necrotic cell death is TNFα-dependent necroptosis involving a microfilament like complex orchestrated by phosphorylated receptor-interacting protein kinase 1 (RIPK1), RIPK3, and MLKL^23^. However, there are conflicting data regarding the role of this cell death mechanism in the pathogenesis of *Mtb*^10,15,24,25^. Since corticosteroids have been shown to interfere with TNFα signaling, we investigated whether specific inhibition of the TNF-receptor (TNFR), TNFα itself, or RIPK1 impacts on susceptibility of cells upon *Mtb* infection. Interestingly, chemical TNFα inhibition using thalidomide, etanercept, or adalimumab as well as RIPK1 inhibition with necrostatins (Nec-1 and Nec-5) failed to protect *Mtb*-infected host cells **(**Fig. 4d; Supplementary Fig. 7**)**. Infected bone-marrow-derived monocytes (BMDM) from *Mlkl*^*−/−*^ mice^26^ were as equally susceptible to *Mtb*-dependent cell death as wild-type cells (Fig. 4e). Similar results were obtained in BMDM derived from *Tnfr1*^*−/−*^ mice^27^ (Fig. 4f). Of note, chemical inhibition of p38 MAPK retained its cytoprotective activity in these knockout cells (Supplementary Fig. 8). These findings separate corticosteroid and p38 MAPK function from TNFα and necroptosis. Recent data indicate that certain triggers of necroptosis require caspase inhibition for execution of programmed cell death^28–30^. A pro-necroptotic milieu can be generated by chemical caspase inhibition, which renders cells susceptible to necrostatin-1 blockade resulting in cell survival. To rule out that this effect occurs in *Mtb*-infected cells, we treated human lung fibroblasts with both Z-VAD-FMK and necrostatin-1. This dual treatment had no cytoprotective effect despite caspase activation after *Mtb* infection (Figs. 4g and 3a, b).

### Host cell protection involves mitochondrial membrane permeability transition

**Fig. 5:**
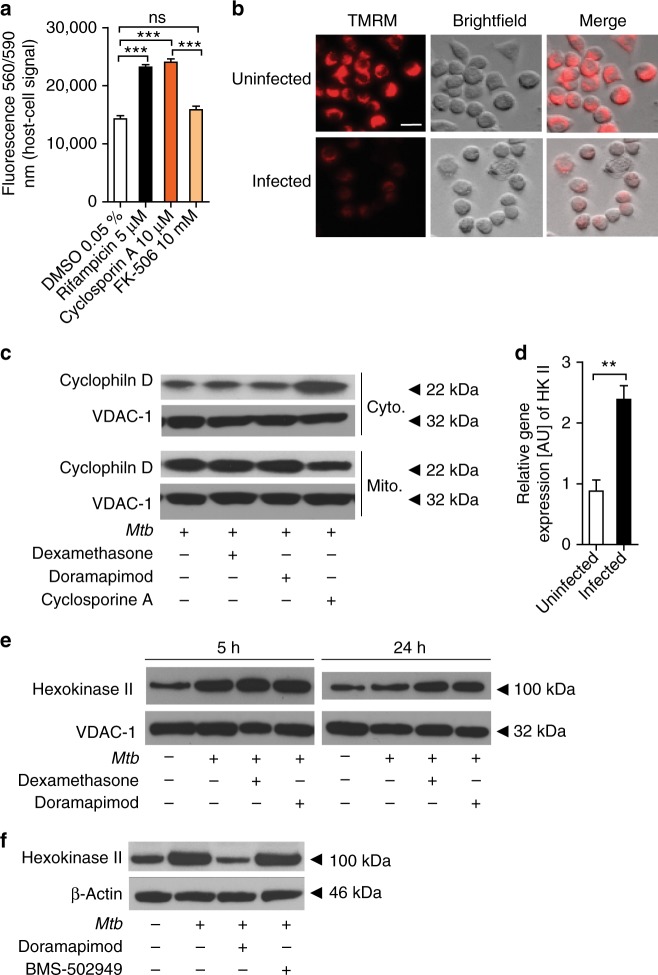
*Mtb* triggers p38 MAPK-dependent necrosis by opening of the mitochondrial permeability transition pore (mPTP). **a** Protective effect of cyclosporine A (CsA; 10 µM) in *Mtb-* infected MRC-5 lung fibroblasts (MOI 10). Viability was quantified 72 h after infection using Prestoblue. **b** Fluorescence microscopy of uninfected and infected (MOI 5) J774.2 Mφ stained with tetramethylrhodamine (TMRM) 48 h post infection (scale bar: 100 µm). Images are representative of two independent experiments. **c** Quantification of cytosolic and mitochondrial CypD following *Mtb* infection. MRC-5 lung fibroblasts were infected with *Mtb* (MOI 10), treated with dexamethasone (5 µM), doramapimod (10 µM), or CsA (10 µM) and lysed for the isolation of mitochondria 5 h post infection. Equal amounts of protein from the mitochondrial and cytosolic fractions were subjected to western blot analysis and the levels of CypD were measured. Voltage-dependent anion-selective channel 1 (VDAC-1) was used as a loading control. **d** Expression of hexokinase II (HKII) in *Mtb*- infected MRC-5 lung fibroblasts. Lysates were obtained 24 h after infection and were analyzed by qRT-PCR. **e** Quantificiation of mitochondrial HKII following *Mtb* infection. J774.2 Mφ were infected with *Mtb*, treated with dexamethasone (5 µM) or doramapimod (10 µM), and mitochondria were isolated 5 and 24 h post infection. Equal amounts of protein were subjected to western blot analysis and the levels of HKII were measured. VDAC-1 was used as a loading control. **f** Quantification of HKII in the cytosol of MRC-5 lung fibroblasts. Whole-cell lysates were obtained from infected untreated cells and from infected cells treated with doramapimod (10 µM) or BMS-582949 (10 µM) 48 h post infection. HKII was quantified by western blot analysis using β-actin as a loading control. Images are representative of two individual experiments. Representative data from at least two experiments with multiple replicates are shown in **a** and **d**. Results are expressed as mean ± SEM and experiments were analyzed using unpaired *t*-test (ns not significant; **p ≤ 0.01; ***p ≤ 0.001)

**Fig. 6:**
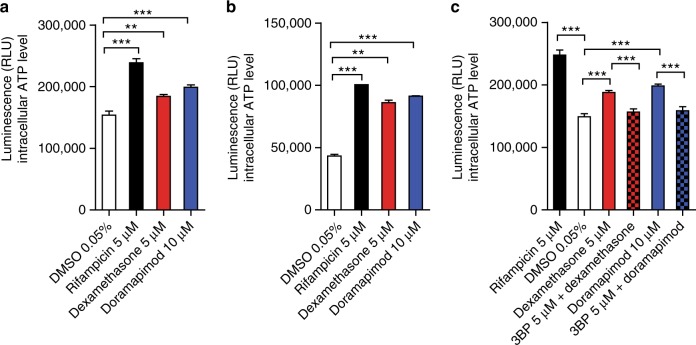
p38 MAPK inhibition preserves intracellular ATP levels. **a,** **b** Intracellular ATP levels in *Mtb*-infected J774.2 Mφ (**a**) and MRC-5 lung fibroblasts (**b**) following 24 and 48 h of infection, respectively. Cells were treated with dexamethasone (5 µM) or doramapimod (10 µM). **c** Intracellular ATP levels in *Mtb*-infected J774.2 Mφ treated with dexamethasone (5 µM) and bromopyruvic acid (3BP) (5 µM) or doramapimod (10 µM) and bromopyruvic acid (5 µM) after 24 h of infection. Data from two experiments with multiple replicates are shown in **a** and **b** and data from one of two experiments are shown in **c**. Results are expressed as mean ± SEM and experiments were analyzed using unpaired *t*-test (**p ≤ 0.01; ***p ≤ 0.001)

Having established that necroptosis is an unlikely mechanism of p38 MAPK-driven cell death, we investigated alternative causes of regulated necrosis. In-depth evaluation of data derived from our high-throughput chemical genetic screen selecting for compounds that abrogate *Mtb*-dependent cell death identified Cyclosporin A (CsA) as a highly cytoprotective hit compound (Supplementary Table 1). CsA inhibits opening of the mPTP by interacting with the mPTP-regulating protein cyclophilin D (CypD)^23^. CsA, but not FK506, a calcineurin inhibitor with no activity on CypD, was as protective as dexamethasone or doramapimod in our host cell survival assays indicating that mitochondrial membrane permeability transition (MPT) is required for *Mtb*-induced cell death (Fig. 5a). Prolonged mPTP opening results in loss of mitochondrial membrane potential (Δ*Ψ*m), mitochondrial rupture, and necrotic cell death^31^. This effect was visualized using tetramethylrhodamine ethyl ester (TMRM) staining which detects Δ*Ψ*m changes. The cell permeable TMRM dye accumulated in healthy, non-infected Mφ but not in infected Mφ giving further proof that *Mtb* kills host cells by mPTP opening which results in disruption of the mitochondrial membrane potential (Fig. 5b). Having identified CypD and the mPTP as a potential target of p38 MAPK-dependent host cell death, we speculated that chemical p38 MAPK inhibition might reduce mitochondrial CypD in infected cells. However, using CsA as a control, we did not observe decreased CypD levels in doramapimod or dexamethasone treated and *Mtb*-infected cells (Fig. 5c; Supplementary Fig. 9). Another important regulator of mPTP opening is the glycolytic enzyme hexokinase II (HKII). While not directly involved in pore formation, translocation and increased binding of HKII to mitochondria protects cells from stress induced by mPTP opening^32^. Relative gene expression analysis showed that HKII is significantly up-regulated 5 h after *Mtb* infection (Fig. 5d). At this early time-point of infection, mitochondrial HKII levels were higher in infected versus non-infected J774 Mφ (Fig. 5e; Supplementary Fig. 10). However, at 24 h post infection HKII levels declined indicating that infected cells are not capable of maintaining protective HKII levels on the mitochondrial membrane (Fig. 5e). Intriguingly, dexamethasone and doramapimod treatment led to sustainably increased levels of mitochondrial HKII in infected cells (Fig. 5e). In human lung fibroblasts we observed a strong increase of cytosolic HKII 48 h after infection indicating significant HKII dissociation from the mitochondrion (Fig. 5f; Supplementary Fig. 11). This effect was fully abrogated in doramapimod-treated cells but not in cells treated with BMS-582949, the substance that fails to dephosphorylate p38 MAPK after *Mtb* infection (Fig. 5f). Mitochondrial HKII ensures tight coupling of glucose phosphorylation and ATP generation^32^. Parallel to mitochondrial dissociation of HKII, we observed a drastic decline of intracellular ATP levels after *Mtb* infection of different host cell types (Fig. 6a, b). This effect was reversed in dexamethasone and doramapimod treated cells (Fig. 6a, b). Co-treatment of *Mtb*-infected J774 Mφ with 3-bromopyruvate (3BP), an HKII inhibitor, abrogated the ATP-sparing effect of dexamethasone and doramapimod which links p38 MAPK activity to HKII (Fig. 6c).

**Fig. 7:**
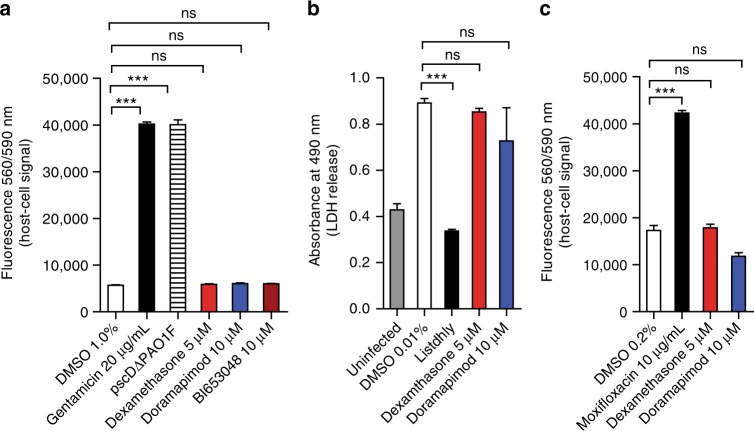
p38 MAPK inhibition fails to protect host cells infected with other bacteria. **a** Effect of dexamethasone (5 µM) and doramapimod (10 µM) on *Pseudomonas aeruginosa* (strain PAO1F)-infected (MOI 0.5) A549 human lung epithelial carcinoma cells. Viability of A549 cells was quantified 24 h post infection using resazurin. A ΔpscC mutant deficient in secretion of T3SS effector proteins was used as a control. **b** Dexamethasone (5 µM) and doramapimod (10 µM) have no cytoprotective effect in BMDM infected with *Listeria monocytogenes* (MOI 2). Host cell viability was quantified 20 h post infection by measuring LDH in the culture medium of the infected cells. A *L. monocytogenes* mutant strain (Listdhly) lacking the *hly* gene, which encodes the virulence factor listeriolysin O, was used as a control. **c** *Salmonella typhimurium*-infected J774.2 Mφ (MOI 3) were treated with dexamethasone (5 µM) and doramapimod (10 µM). Host cell viability was quantified 48 h post infection using resazurin. Data from one of two experiments are shown in **a** and **c**; data from two experiments with multiple replicates are shown in **b**. Results are expressed as mean ± SEM and experiments were analyzed using unpaired *t*-test (ns not significant; ***p ≤ 0.001)

The targeted p38 MAPK controlled pathway we identified seems to be specific for the intracellular pathogen *Mtb* since type III secretion system-mediated host cell death caused by Gram-negative pathogens, such as Pseu*domonas aeruginosa* and *Salmonella typhimurium*, could not be blocked under similar conditions (Fig. 7a, c). The same holds true for protection of macrophages infected with the intracellular pathogen *Listeria monocytogenes* (Fig. 7b). Though being potent inhibitors of *Mtb*-induced necrosis, none of the described substances led to a change of the intracellular mycobacterial load of J774 Mφ compared to untreated control cells (Supplementary Fig. 12). This elucidates that phagocytosis or intracellular replication of mycobacteria is not impaired by host cell pretreatment with the respective drugs. A finding which also explains why the potent necrosis inhibitors we identified were missed in other phagocyte-based drug screens using intracellular mycobacterial replication but not host cell survival as primary readout^33^.

### Doramapimod treatment of mice is cytoprotective for infected Mφ

**Fig. 8:**
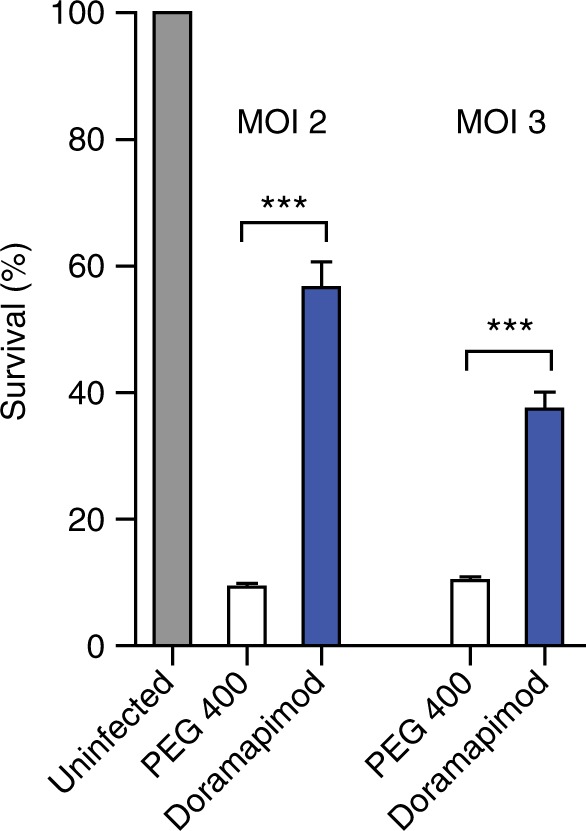
Doramapimod treatment of mice is protective for peritoneal macrophages. Treatment of C57BL/6 mice with doramapimod is protective for *Mtb-*infected peritoneal Mφ (PM). Mice received doramapimod (100 mg/kg q.d.; i.p.) for 3 days. PM were isolated by CD11b^+^ selection and infected with *Mtb* (MOI 2 or MOI 3). Cell survival was assessed 24 h after infection by DAPI staining, using uninfected PM as a control. Control mice received the vehicle PEG 400. Data from one experiment with three mice per group and multiple replicates are shown. Results are expressed as mean ± SEM and experiments were analyzed using unpaired *t*-test (***p ≤ 0.001)

To determine whether in vivo chemical p38 MAPK inhibition has the potential to reduce necrotic cell death in mouse Mφ, we performed an experiment in which we treated C57BL/6 mice with doramapimod (100 mg/kg (q.d.; i.p.) for 3 days. The following day, CD11b^+^ peritoneal Mφ were isolated using labeled magnetic microbeads and infected with *Mtb* at two different MOI. Twenty-four hours after infection, cell death was quantified by fluorescent staining. Mφ isolated from doramapimod-treated mice were strongly protected from *Mtb*-induced cell death in contrast to cells from mice receiving the vehicle only (Fig. 8). This finding highlights the potential of chemical p38 MAPK inhibition as a therapeutic option to abrogate necrotic cell death caused by *Mtb* infection. In addition, the experiment rules out a direct inhibitory effect of doramapimod on *Mtb* since Mφ infection was performed after in vivo treatment and extensive washing of CD11b^+^ selected monocytes.

## Discussion

**Fig. 9:**
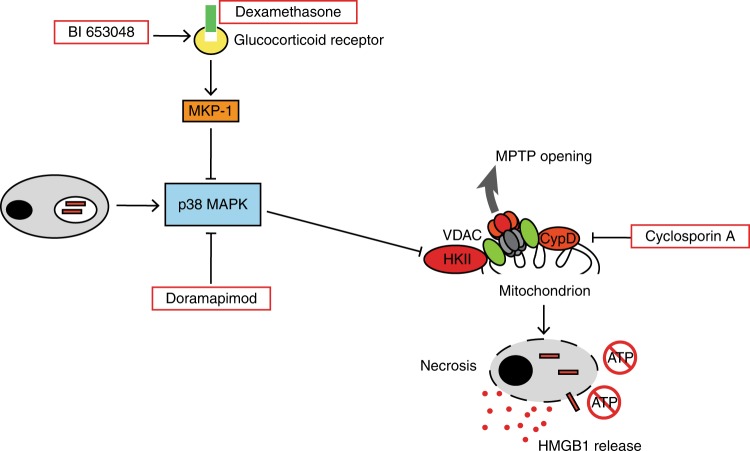
Model depicting a novel mode of *Mtb-*induced cell death and its chemical inhibition. Phagocytosis of *Mtb* leads to p38 MAPK phosphorylation and hexokinase II (HKII) dissociation, which seems to open the mitochondrial permeability transition pore (mPTP) subsequently leading to necrotic cell death which can be monitored by high-mobility group protein B1 (HMGB1) release. Several substances interfere with this pathway to protect cells. Dexamethasone or BI653048 treatment inhibits p38 MAPK phosphorylation via the glucocorticoid receptor (GR) and MAPK phosphatase 1 (MKP-1). Direct p38 MAPK blockade using doramapimod has a similar cytoprotective effect. p38 MAPK promotes dissociation of hexokinase II (HKII) from the mitochondrion leading to ATP depletion. Cyclosporin A directly interferes with mPTP opening via inhibition of cyclophillin D, a regulator of the mPTP. *VDAC* voltage-dependent anion channel

Here, by exploiting a high-throughput chemical genetics approach, we show that corticosteroids possess a so far undescribed TNFα-independent inhibitory effect on necrotic cell death. Our detailed analysis revealed a highly druggable process involving the GR, MKP-1, and p38 MAPK, which ultimately impacts on *Mtb*-induced necrosis by provoking mitochondrial MPT and loss of mitochondrial integrity (Fig. 9). Opening of the mPTP is an emerging mechanism of regulated necrosis which becomes increasingly associated with pathogenesis of several diseases^23^. However, the exact molecular composition of the channel forming the mPTP is a matter of debate. Candidate molecules include the adenine nucleotide translocase, the voltage-dependent anion channel (VDAC), and components of the ATP synthase^31^. A confirmed regulatory component of the pore complex is CypD^31^. Accordingly, chemical CypD inhibition using CsA is a well-known mechanism to inhibit mPTP opening and has been previously shown to block *Mtb*-induced cell death^10,25^. However, CsA is a non-selective cyclophylin inhibitor leading to immunosuppression by blocking calcineurin activity in T cells. Exploiting chemical mPTP inhibition as HDT requires either selective CypD inhibitors or substances that target alternative components of the complex. Here we showed that chemical activation of the GR or p38 MAPK inhibition does not impact on mitochondrial CypD but interferes with mitochondrial HKII function. HKII is primarily associated with the mitochondrial membrane where it binds VDAC and CypD^32^. The interaction of HKII with mitochondria represents an intrinsic molecular mechanism to protect cells against stress cues ultimately leading to necrotic death. Mitochondrial HKII has the ability to inhibit reactive oxygen species generation at mitochondria which prevents opening of the mPTP^32,34^. Subsequently, a decrease in HKII levels sensitizes cells to necrotic stimuli^32^. However, little is known on regulatory aspects of HKII transcription, post-translational modification, and mitochondrial translocation. Here we provide indirect evidence that the activation of p38 MAPK by intracellular *Mtb* initiates mitochondrial dissociation of HKII, which, in turn, promotes cell death via mitochondrial permeability transition and ATP depletion. Interestingly, it was recently shown that pathogen-driven HKII dissociation represents an important means of immune sensing in other Gram-positive bacteria^35^. Thus, HKII seems to be a common bacterial target in host–pathogen interactions of Gram-positive organisms, although the underlying molecular mechanisms still need to be determined. Our data showing that in *Mtb*-infected cells, HKII dissociation can be abrogated by MKP-1 activation (dexamethasone, BI653048) and p38 MAPK inhibition represent a possible molecular link to further elucidate the regulatory mechanisms of HKII.

p38 MAPK is a validated target in autoimmune and inflammatory diseases and several small-molecule inhibitors have been tested in clinical trials mainly against rheumatoid arthritis, psoriasis, and Crohn’s disease^36^. Histopathological investigation of human biopsies revealed p38 MAPK phosphorylation in Mφ surrounding TB-granulomas, indicating that this kinase is engaged and may be a potential target for HDT in TB^37^. We were surprised to identify differing activity of several commercially available and clinically tested p38 MAPK inhibitors. All tested substances are highly specific for p38 MAPK and display an IC_50_ in the nanomolar range^36^. However, among the tested substances there are differences with regard to inhibition of the p38 MAPK isotypes α, β, γ, and δ. Doramapimod is a broad spectrum inhibitor potently targeting all four isotypes whereas pamapimod is much more selective for the α and β isotypes. In addition, the reason for doramapimod being the sole inhibitor that maintained low phosphorylation levels of p38 MAPK at later time points of infection may also be found in its unique binding mechanism on the target kinase^38^. Our data showing protection of Mφ after p38 MAPK gene knock down confirm dephosphorylation and activity data obtained using doramapimod and verify the key role of this kinase in *Mtb*-induced cell death.

It is unlikely that p38 MAPK directly interacts with mitochondrial bound HKII and identification of the linking molecule requires further research. A starting point could be p53, a regulatory protein which shuttles between different cell compartments and physically interacts with the mitochondrial membrane^39^. We have generated preliminary data indicating that chemical p53 inhibition in *Mtb*-infected cells has similar effects as p38 MAPK inhibition with regard to cytoprotection and ATP depletion (Supplementary Fig. 13). In the past decade, numerous reports have highlighted the crucial role of p53 in host–pathogen interactions^40^. p53 is known to influence mPTP either via its own mitochondrial translocation or by indirect modulation of mPTP components including HKII^39,41–43^. In addition, p38 MAPK-mediated p53 phosphorylation constitutes a critical step in pathogen-driven cell death and chemotherapeutic killing of cancer cells^44,45^. This possible link in *Mtb* pathogenesis requires a more detailed examination in the future.

By using genetic and chemical targeting, we provide clear evidence that TNFα and RIPK1/MLKL-driven necroptosis is not involved in corticosteroid- and p38 MAPK-dependent mitochondrial depolarization in *Mtb*-infected cells. Two studies linked RIP-kinase signaling to mitochondrial membrane depolarization and cell death in TB^10,25^. Our contradictory findings confirm and further extend results of a recent study on *Mlkl*^*−/−*^ mice which presented lung histopathology and mycobacterial burdens indistinguishable from control mice^15^. Collectively, these data show that necroptotic signaling is unlikely a trigger for mPTP opening in TB. In addition, our findings on TNFα-independent host-protective effects of corticosteroids may also explain clinical data showing that adjuvant corticosteroid treatment is more beneficial for TB patients than adjuvant treatment with specific TNFα inhibitors^46^. Our findings have immediate consequences for future HDT trials targeting primarily TNFα.

In summary, our report indicates that *Mtb* infection leads to phosphorylation of p38 MAPK and mitochondrial HKII dissociation, which mediates cell death via mitochondrial permeability transition and ATP depletion. A mechanism which can be disrupted by MKP-1 activation (dexamethasone, BI653048) and p38 MAPK inhibition (Fig. 9). These findings may have the potential to extend the portfolio of host-directed anti-TB therapeutics. Finally, we provide proof of concept for readout switching from bacterial growth inhibition to host cell survival, which can identify inhibitors of *Mtb*-induced necrotic host cell death in future high-throughput drug screens.

## Methods

### Ethics statement

All animal experiments were performed in accordance with institutional, state, and federal guidelines (approved by the Landesamt fuer Natur, Umwelt und Verbraucherschutz (LANUV) North Rhine-Westphalia, Germany).

Blood samples were obtained from patients with active TB before therapy was initiated and from healthy volunteers. The study was approved by the University of Cologne Ethics Committee (18-079). Patients as well as healthy volunteers participated after giving written informed consent.

### Drugs used in this study

Rifampicin was purchased from AppliChem. Bromopyruvic acid, dexamethasone, gentamicin, staurosporine, and thalidomide were purchased from Sigma-Aldrich. Cyclosporin A, doramapimod, moxifloxacin, necrostatin-1, necrostatin-5, and tacrolimus (FK506) were obtained from Cayman Chemicals. BMS-582949, pamapimod, and Z-VAD-FMK were purchased from Selleckchem. Puromycin dihydrochloride was from Carl Roth (Germany). GR agonist BI653048 was provided by the opnMe platform (Boehringer Ingelheim). The Prestwick chemical library was used for high-throughput screening.

### Mice

Six- to eight-week-old female C57BL/6 mice were bred in-house and used at an age of 8–12 weeks. Animals were housed under specific pathogen-free conditions in the Laboratory Animal Facility of the University Hospital Cologne.

### Isolation of human macrophages

Human mononuclear leukocytes were isolated from the blood of healthy donors or TB patients and monocytes were obtained by magnetic-activated cell sorting (MACS) of CD14^+^ cells using CD14 MicroBeads (Miltenyi Biotec). Monocytes were differentiated into Mφ for 4 days in Roswell Park Memorial Institute 1640 (RPMI) medium supplemented with 10% fetal bovine serum (FBS) and 50 ng/ml human macrophage colony-stimulating factor (Miltenyi Biotec) at a density of 1 × 10^5^ cells per well in 96-well plates (Corning). Before infecting primary Mφ with varying MOI (1 or 2) of *Mtb*, the growth media was changed to RPMI supplemented with 10% FBS. Mφ were grown at 37 °C with 5% CO_2_.

### Isolation of mouse macrophages

Bone-marrow-derived Mφ (BMDM) were isolated as previously described^47^. Briefly, mice were sacrificed by cervical dislocation, the legs were removed, and dissected from adherent tissue. Femurs and tibia were cut off at both ends and the bone marrow was expelled with RPMI using a 27g needle. After centrifugation for 10 min at 400 × *g* cells were plated in Petri dishes and incubated at 37 °C and 5% CO_2_. Bone marrow cells were differentiated in VLE RPMI 1640 (Biochrom) supplemented with 10% FCS (Biowest), 10 mM HEPES (Biochrom), 10 µg/ml penicillin/streptomycin (Biochrom), 1 mM sodium pyruvate (Biochrom), 2mM l-glutamine (Biochrom), and 15% M-CSF (supernatants of L929 mouse fibroblasts), for 7 days. Five days after start of the culture fresh medium was added.

Peritoneal Mφ (PM) were isolated from C57BL/6 mice by peritoneal lavage and subsequent CD11b^+^ selection by MACS using CD11b MicroBeads (Miltenyi Biotec). For ex vivo experiments, mice received doramapimod (100 mg/kg, in polyethylene glycol 400 (PEG 400) i.p.) for 3 days and control mice received PEG 400 only. PM were isolated and seeded in 96-well plates at a density of 5 × 10^4^ cells per well in Dulbecco’s modified Eagle's medium (DMEM) supplemented with 10% FBS. Following adherence of the cells, PM were infected with *Mtb* (MOI 2 or 3) for 24 h and analyzed by fluorescence microscopy.

### Cell culture

MRC-5 human lung fibroblasts were provided by the Coriell Institute for Medical Research and cultured in minimum essential medium (MEM) supplemented with 10% heat-inactivated FBS(PAN-Biotech), 1% non-essential amino acids, and 1% sodium pyruvate. J774A.1 Mφ and J774.2 Mφ (ATCC) were grown in DMEM supplemented with 10% FBS. BV-2 microglia were grown in DMEM supplemented with 10% FBS and 1% sodium pyruvate. A549 human lung epithelial carcinoma cells (ATCC) were grown in RPMI medium supplemented with 10% FBS.

### Culture conditions of bacteria

The mycobacterial strain Erdman (provided by S.T. Cole) was grown in Middlebrook 7H9 broth, supplemented with 10% albumin dextrose catalase (ADC), 0.05% Tween-80 and 0.2% glycerol, or 7H10 agar plates, supplemented with 10% ADC and 0.5% glycerol. All *P. aeruginosa* strains (PAO1F WT and PAO1F ΔpscD) were routinely grown in 2xYT medium and all *S. typhimurium* strains as well as *L. monocytogenes* strains were grown in brain heart infusion medium.

### Survival assays

For the survival assays, compounds were preplated into 96-well plates at different concentrations. MRC-5, J774.2 Mφ, or A549 cells were harvested and seeded at a density of 2 × 10^4^ cells per well and were allowed to adhere for 3 h. Then cells were infected with *Mtb* Erdman with varying MOI.

For high-throughput screening, MRC-5 lung fibroblasts were infected with an MOI of 10 and were analyzed 72 h post infection using the fibroblast survival assay as previously described^19,20^. In brief, 4000 MRC-5 cells were seeded in 384-well cell bind plates (Corning). After 3 h at 37 °C, cells were infected with *Mtb* Erdman resuspended in MEM medium. After the 72 h incubation period, survival of fibroblasts was determined by addition of PrestoBlue (10% final concentration) and fluorescence reading in a Cytation 3 Cell Imaging Multi-Mode Reader (Biotek).

For the survival assay with *P. aeruginosa*, A549 cells were seeded in a 96- well plate containing different drugs. Following adherence, cells were infected with *P. aeruginosa* at an MOI of 0.5 and after 4 h moxifloxacin was added at a final concentration of 10 μM. The next day, 10% resazurin was added and fluorescence was detected using a Tecan Safire II fluorescence reader.

For the survival assay with *S. typhimurium*, J774.2 Mφ were infected at an MOI of 2 with *S. typhimurium* for 3 h, before adding moxifloxacin at a final concentration of 10 μM. After 48 h of incubation, 10% resazurin was added and fluorescence was detected using a Tecan Safire II fluorescence reader.

For the survival assay with *L. monocytogenes*, BMDM were seeded at a density of 8 × 10^4^ cells per well in a 96-well plate and differentiated into macrophages for 7 days^47^. BMDM were then infected with *L. monocytogenes* at an MOI of 2 for 2 h, before adding moxifloxacin at a final concentration of 10 μM. The following day, LDH was measured in the supernatant of the cells.

### Fluorescence microscopy

For fluorescence microscopy J774 Mφ and BV-2 microglia were seeded at a density of 2 × 10^4^ cells per well in a 96-well plate. Following adherence, cells were infected with *Mtb* Erdman at an MOI of 5 for 48 h. Isolated primary human monocytes were seeded at a density of 1 × 10^5^ cells per well and BMDM were seeded at a density of 8 × 10^4^ cells per well in a 96-well plate. After differentiation of monocytes into Mφ, cells were infected with *Mtb* for 48 h. PM were seeded at a density of 5 × 10^4^ cells per well in a 96-well plate and infected with *Mtb* at an MOI of 2 or 3 for 24 h. Afterwards, cells were washed several times with PBS and fixed with 4% paraformaldehyde/PBS. To quantify the number of surviving cells, cells were stained with 4′,6-diamidino-2-phenylindole (DAPI; Thermo Fisher Scientific) and images were acquired on an IX81 inverted microscope (Olympus) using cellSens standard software (Olympus) as well as Fiji processing software.

### CFU determination

J774.2 Mφ were seeded one day prior to the infection in 96-well plates at a density of 5 × 10^3^ cells per well. Cells were pre-treated with different drugs for 2 h and infected with *Mtb* Erdman at an MOI of 2. The following day, cells were washed several times with PBS to remove unphagocytosed bacteria and fresh medium containing compounds or DMSO was added. Plates were sealed and incubated at 37 °C in 5% CO_2_. Five days post infection cells were lysed with 0.1% sodium dodecyl sulfate (SDS). Viable bacteria were grown in serial dilutions on 7H10 agar plates. Colonies were counted after 10–14 days of incubation at 37 °C.

### HMGB1 ELISA

The release of HMGB1 from cells was detected by using a HMGB1 ELISA kit (IBL International). Briefly, 1 × 10^6^ MRC-5 lung fibroblasts were seeded in a six-well plate and the following day cells were infected with *Mtb* Erdman at an MOI of 10. Infected cells were incubated for 48 h at 37 °C in 5% CO_2_ and the supernatant was collected. The samples were transferred into a 96-well plate pre-coated with polyclonal anti-HMGB1 and incubated for 24 h at 37 °C. Afterwards, HMGB1 conjugated to peroxidase was added to the samples and the plate was incubated for another 2 h at 25 °C. For detection, TMB substrate solution was used. The absorbance was measured and analyzed using a Multiskan™ FC Microplate Photometer with internal software (Thermo Fisher Scientific).

### Caspase assay

Caspase activity was measured with the Caspase-Glo^®^ 3/7 Assay according to the manufacturer’s instructions (Promega). Cells were harvested and seeded at a density of 2 × 10^4^ cells per well in a 96-well plate. Following adherence of the cells, MRC-5 lung fibroblasts (MOI 10) and J774.2 Mφ (MOI 5) were infected with *Mtb* Erdman and incubated for up to 48 h at 37 °C in 5% CO_2_. The Caspase- Glo^®^ Reagent was added to the wells 24 and 48 h post infection and luminescence was measured in a BioTek™ Cytation™ 3 Cell Imaging Multi-Mode Reader.

### LDH release assay

The Pierce™ LDH Cytotoxicity Assay Kit (Thermo Fisher Scientific) was conducted according to the manufacturer’s recommendations. Harvested MRC-5 lung fibroblasts and J774.2 Mφ were seeded in a 96-well plate (2 × 10^4^ cells per well) and infected with *Mtb* Erdman at an MOI of 10 and 5, respectively. Twenty-four, 48 and 72 h post infection the release of LDH into the supernatant of cells was analyzed by measuring the reduction of tetrazolium salt to a red formazan product in a BioTek™ Cytation™ 3 Cell Imaging Multi-Mode Reader (Biotek). The reaction mixture was added to the samples at a ratio of 1:1 and the plates were incubated for 30 min at room temperature. Lysed cells were used as maximum LDH activity control and untreated cells were used as spontaneous LDH release control. For primary human Mφ, 1 × 10^5^ cells per well were infected with an MOI of 1 or 2 with *Mtb* and the supernatant was collected 24 and 48 h post infection to measure the release of LDH.

### ATP assay

The CellTiter-Glo^®^ 2.0 Assay (Promega) was conducted according to the manufacturer’s recommendations. Harvested MRC-5 lung fibroblasts and J774.2 Mφ were seeded in a 96-well plate (2 × 10^4^ cells per well) and infected with *Mtb* Erdman at an MOI of 10 and 5, respectively. Twenty-four and 48 h post infection the amount of ATP was quantified by adding the CellTiter-Glo^®^ 2.0 reagent to the samples at a ratio of 1:1 and incubating the plate for 10 min at room temperature. Afterwards luminescence was detected in a BioTek™ Cytation™ 3 Cell Imaging Multi-Mode Reader.

### Isolation of mitochondria

For the isolation of mitochondria from infected MRC-5 lung fibroblasts (MOI 10) and J774.2 Mφ (MOI 5) the Mitochondria Isolation Kit for Cultured Cells (Thermo Fisher Scientific) was used according to the manufacturer’s recommendations. Briefly, 5 and 24 h post infection 1 × 10^7^ cells were washed several times with PBS and detached from the culture dish using a cell scraper. Mitochondria were obtained using the reagent-based method. For separation of the mitochondrial and the cytosolic fraction, the sample was centrifuged at 12,000 × *g* for 15 min at 4 °C. The mitochondria were lysed with 2% CHAPS (Thermo Fisher Scientific) in Tris-buffered saline (TBS; 25 mM Tris, 150 mM NaCl, pH 7.2) containing Halt™ Protease and Phosphatase Inhibitor Cocktail (Thermo Fisher Scientific). Both the mitochondrial and cytosolic fractions were stored at −80 °C and the purity of the mitochondrial fraction was analyzed by western blotting.

### TMRM staining

J774.2 Mφ were seeded in a 96-well plate (5 × 10^3^ per well) and infected with *Mtb* Erdman at an MOI of 5 for varying times (24 and 48 h). For staining with TMRM, cells were washed several times with PBS before adding a final concentration of 100 nM of TMRM (Sigma-Aldrich). Cells were incubated at 37 °C in 5% CO_2_ for 30 min and washed with PBS. Images were acquired on an IX81 inverted microscope (Olympus) using cellSens standard software (Olympus) as well as Fiji processing software.

### Immunoblot analysis

1 × 10^6^ MRC-5 lung fibroblasts or J774.2 Mφ were seeded in 10 cm culture dishes and allowed to adhere for at least 24 h before infection. Whole-cell lysates were obtained 5, 24, 30, and 48 h post infection using radioimmunoprecipitation assay (RIPA) buffer. The protein concentration was measured with the Pierce™ BCA Protein Assay Kit (Thermo Fisher Scientific). Proteins were separated by SDS-polyacrylamide gel electrophoresis and transferred to a PVDF membrane using the Trans-Blot^®^ Turbo™ Transfer System (Bio Rad). The membrane was blocked for 1 h with either 5% dried milk for total proteins or 5% bovine serum albumin for phosphorylated proteins in Tris-HCl buffer (10 mM, pH 7.6), containing NaCl (150 mM) and Tween 20 (1%). Subsequently, the membrane was incubated with primary antibodies at 4 °C overnight. The next day, the blots were incubated for 1 h at room temperature with the corresponding secondary, horseradish peroxidase-conjugated, antibody. Visualization of the transferred proteins was done by using the 20X LumiGLO^®^ Reagent and 20X Peroxide (Cell Signaling Technology) and X-ray films were processed in a Curix 60 (AGFA). All antibodies except anti-cyclophilin D (ab110324; Abcam) were purchased from Cell Signaling Technology and used according to the manufracturer’s recommendations. The following antibodies were used: anti-β-Aktin (4970), anti-p38 MAPK (9212), anti-phospho p38 MAPK (4511), anti-JNK (9252), anti-phospho JNK (4688), anti-ERK (9102), anti-phospho ERK (9101), anti- caspase 3 (9662), anti-cleaved caspase 3 (9661), anti-hexokinase II (2867), and anti-VDAC-1 (4866).

### Quantitative real-time PCR

For the isolation of total RNA, the RNeasy Mini Kit (Qiagen) was used. Briefly, 1 × 10^6^ MRC-5 lung fibroblasts and J774.2 Mφ were seeded in a six-well plate and infected with *Mtb* Erdman at an MOI of 10 and 5, respectively. Five and 24 h post infection, cells were washed several times with PBS, detached from the plate by using a cell scraper, and lysed with RLT buffer containing β-mercaptoethanol. Quantification of RNA was done using a NanoDrop spectrophotometer (Thermo Fisher Scientific); *A*_260_/*A*_280_ ratios of all samples remained within the range of 1.90–2.10. cDNA synthesis was performed with the SuperScript III First-Strand Synthesis SuperMix for qRT-PCR (Thermo Fisher Scientific) and real-time PCR was conducted using Light Cycler Fast Start DNA MasterPLUS SYBR Green Kit (Roche) and specific primers for hexokinase II (fw 5′-TCTAAGCGGTTCCGCA; rev 5′-AGAAGGGTCATACCTGG), MKP-1 (fw 5′-GGAATCTGGGTGCAGT; rev 5′-CTGGTAGTGACCCTCAA), p38 MAPK (fw 5′-GCCCGAACGATACCAG; rev 5′-CTGAAACGGTCTCGACA), GAPDH (fw 5′-GGTATCGTGGAAGGACT; rev 5′-GGGTGTCGCTGTTGAA), and HMBS (fw 5′-TGCACGATCCCGAGAC; rev 5′-CGTGGAATGTTACGAGC). The analysis was done by comparative Cp method and Cp values were normalized against the house-keeping genes GAPDH and HMBS. To eliminate non-specific amplification, a melting curve analysis was performed.

### Generation of a p38 knock-down macrophage cell line

Utilizing the retroviral MSCV-LTRmiR30-PIG (MLP) vector^48^ a p38-specific shRNA based on predictions from siRNA Scales^49^ and RNAi central (http://katahdin.cshl.edu) (Sequence:TGCTGTTGACAGTGAGCGATACCACGATCCTGATGATGAATAGTGAAGCCACAGATGTATTCATCATCAGGATCGTGGTACTGCCTACTGCCTCGGA) was generated. J774A.1 cells were transduced with shP38-MLP and MLP control vector respectively by puromycin selection. Knock-down efficiency was determined by qRT-PCR as described above.

### Statistical analysis and general methods

Data are expressed as mean ± SD or mean ± SEM. The data were analyzed with the Graphpad Prism version 7 software program. Differences were assessed using two-tailed Student t-tests and differences with *p* values of < 0.05 were considered to be statistically significant. Sample size for animal studies was chosen based on prior experience with similar models and published data showing similar experiments. No data were excluded from the analysis of experiments. Mice were commercially sourced and randomized into experimental groups upon arrival, and all animals within a single experiment were processed at the same time, there was no blinding performed. Data display similar variance between groups and are normally distributed where parametric tests have been used.

### Reporting Summary

Further information on experimental design is available in the Nature Research Reporting Summary linked to this article.

## Supplementary information

Supplementary Information

Reporting Summary

## Data Availability

The authors declare that all data supporting the findings of this study are available within the paper and its supplementary information files.
