# Supplementary material for: Corticosteroids inhibit Mycobacterium tuberculosis-induced necrotic host cell death by abrogating mitochondrial membrane permeability transition
Source: Nat Commun. 2019 Feb 8;10:688. doi: 10.1038/s41467-019-08405-9 (PMC6368550; doi:10.1038/s41467-019-08405-9)
Supplement: Supplementary file 1 — Supplementary Information [file 41467_2019_8405_MOESM1_ESM.docx]

**SUPPLEMENTARY INFORMATION FOR**

**Corticosteroids inhibit *Mycobacterium tuberculosis*-induced necrotic host cell death by abrogating mitochondrial membrane permeability transition**

**Gräb et al., 2018**

**Supplementary Figure 1**

**
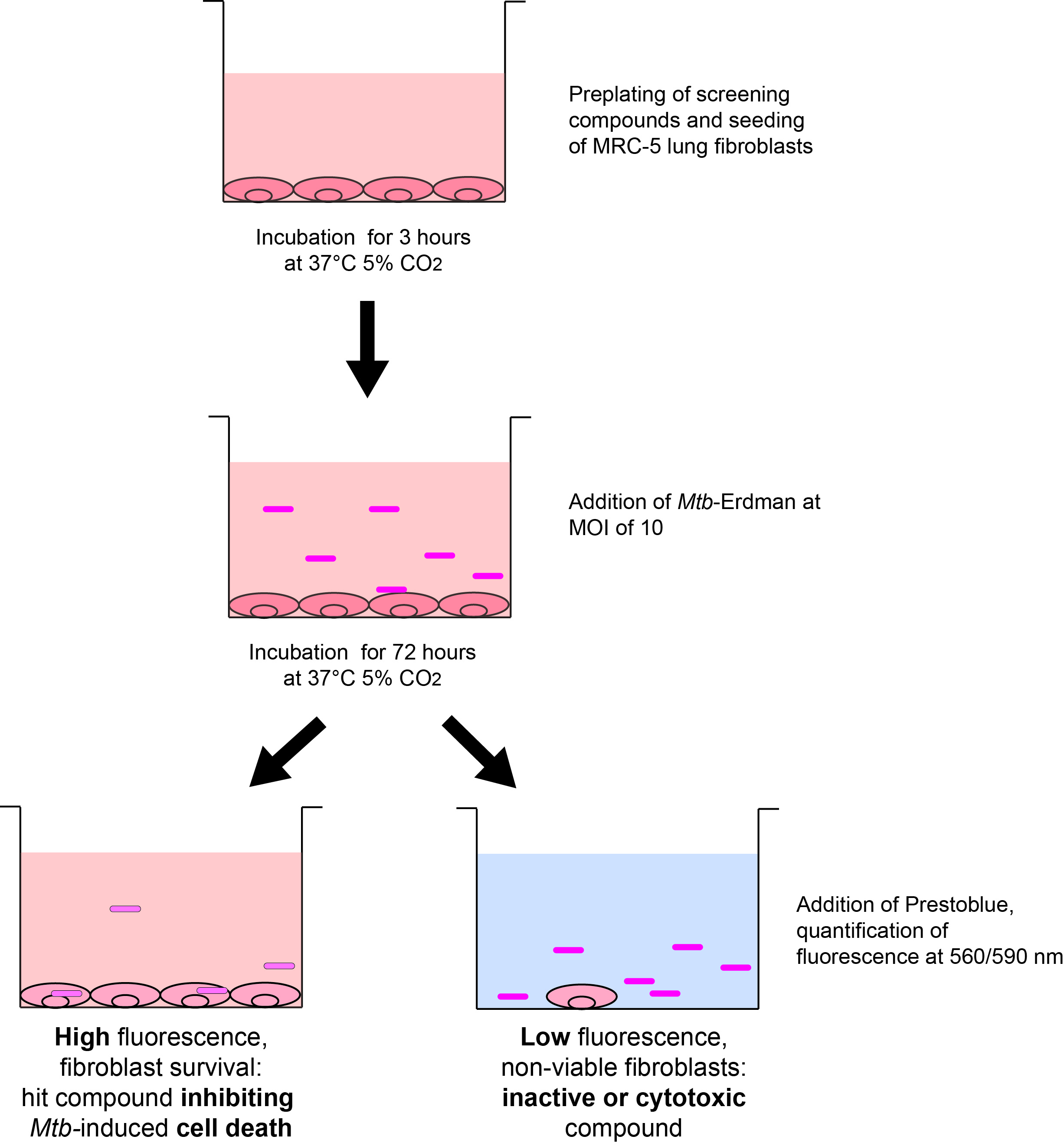
**

**Supplementary Figure 1**. **Flow diagram of the host cell-based HTS used in this study.** Due to the rapid turnover of PrestoBlue by eukaryotic cells, background fluorescence from bacteria is negligible. A full description of the assay can be found in the methods section. MOI: multiplicity of infection (illustration created by J.R.).

**Supplementary Fig. 2**

**
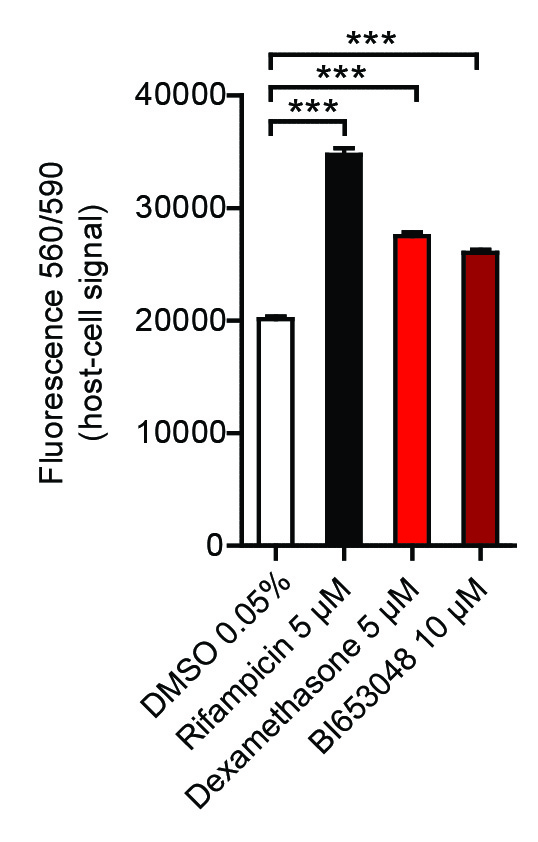
**

**Supplementary Figure 2. Protective effect of the GR agonist BI653048 in MRC-5 lung fibroblasts.** Treatment of *Mtb*-infected MRC-5 lung fibroblasts (MOI 10) with BI653048 (10 µM) reduces mycobacterial cytotoxicity. Viability was quantified using PrestoBlue. Results are expressed as mean ± SEM. Data of one experiment with multiple replicates were analyzed using unpaired t test (***, p≤ 0.001).

**Supplementary Fig. 3**

**
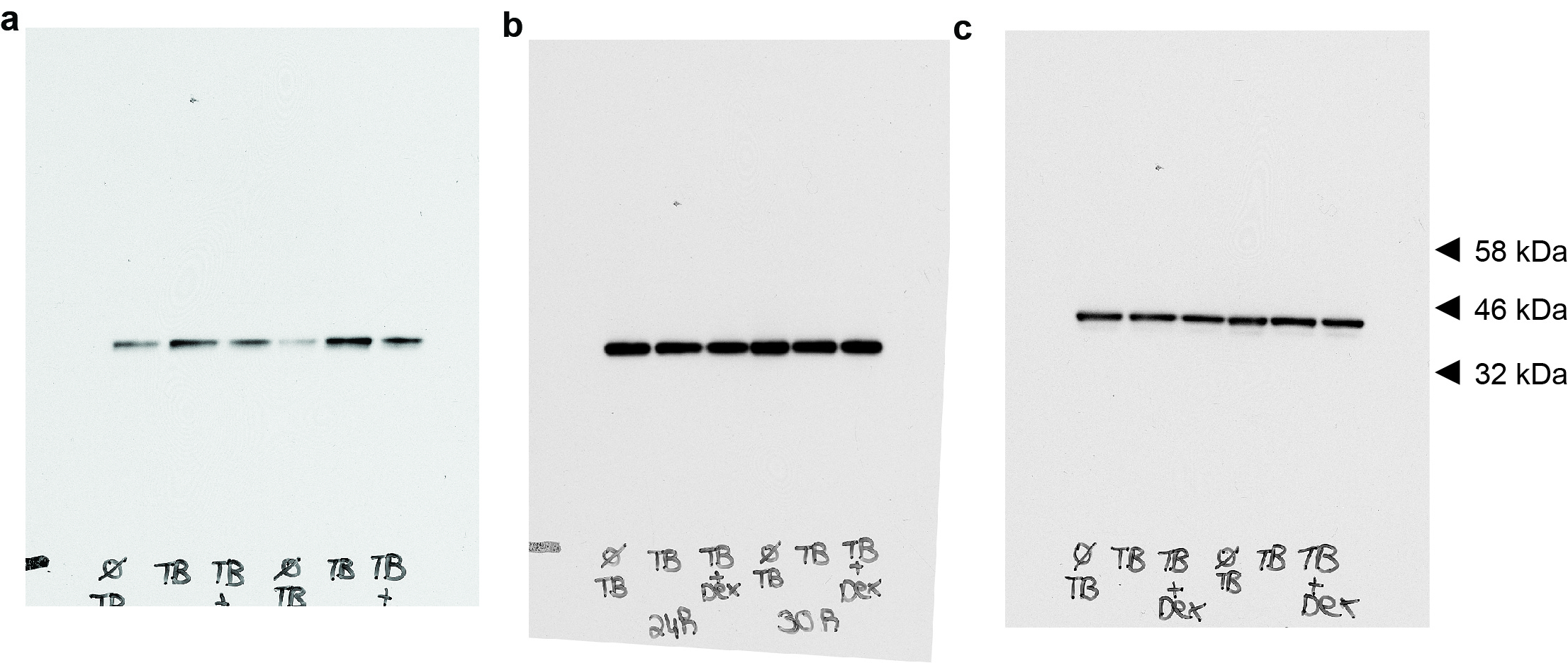
**

**Supplementary Fig. 3. *Mycobacterium tuberculosis* (*Mtb*) induces p38 MAPK activation in J774.2 macrophages (Mφ).** Quantification of phosphorylated p38 MAPK **(a)** and total p38 MAPK **(b)** in uninfected, *Mtb* infected (MOI 5) and dexamethasone (5 µM) treated, *Mtb* infected J774.2 Mφ. Whole cell lysates were obtained 24 h and 30 h post infection and subjected to Western blot analysis, using β-actin as a loading control **(c)**. Blots are representative of three individual experiments.

**Supplementary Fig. 4**

**
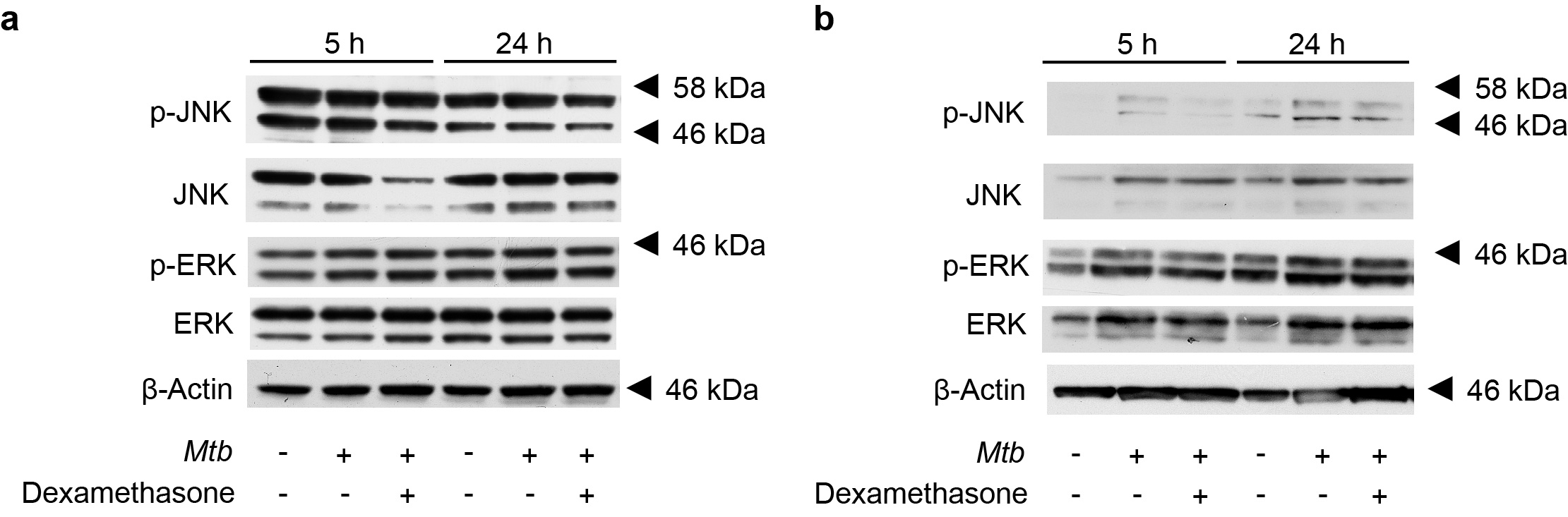
**

**Supplementary Figure 4. *Mtb* infection does not induce extracellular signal–regulated kinase (ERK) or c-Jun N-terminal kinase (JNK) activation.** Whole cell lysates were obtained at 5 h and 24 h from infected J774.2 Mφ **(a)** and MRC-5 lung fibroblasts **(b)** treated with or without dexamethasone (5 µM) and equal amounts of protein were subjected to Western blot analysis to determine the levels of phosphorylated and total ERK and JNK. β- Actin was used as a loading control. Images are representative of two to three individual experiments.

**Supplementary Fig. 5**

**
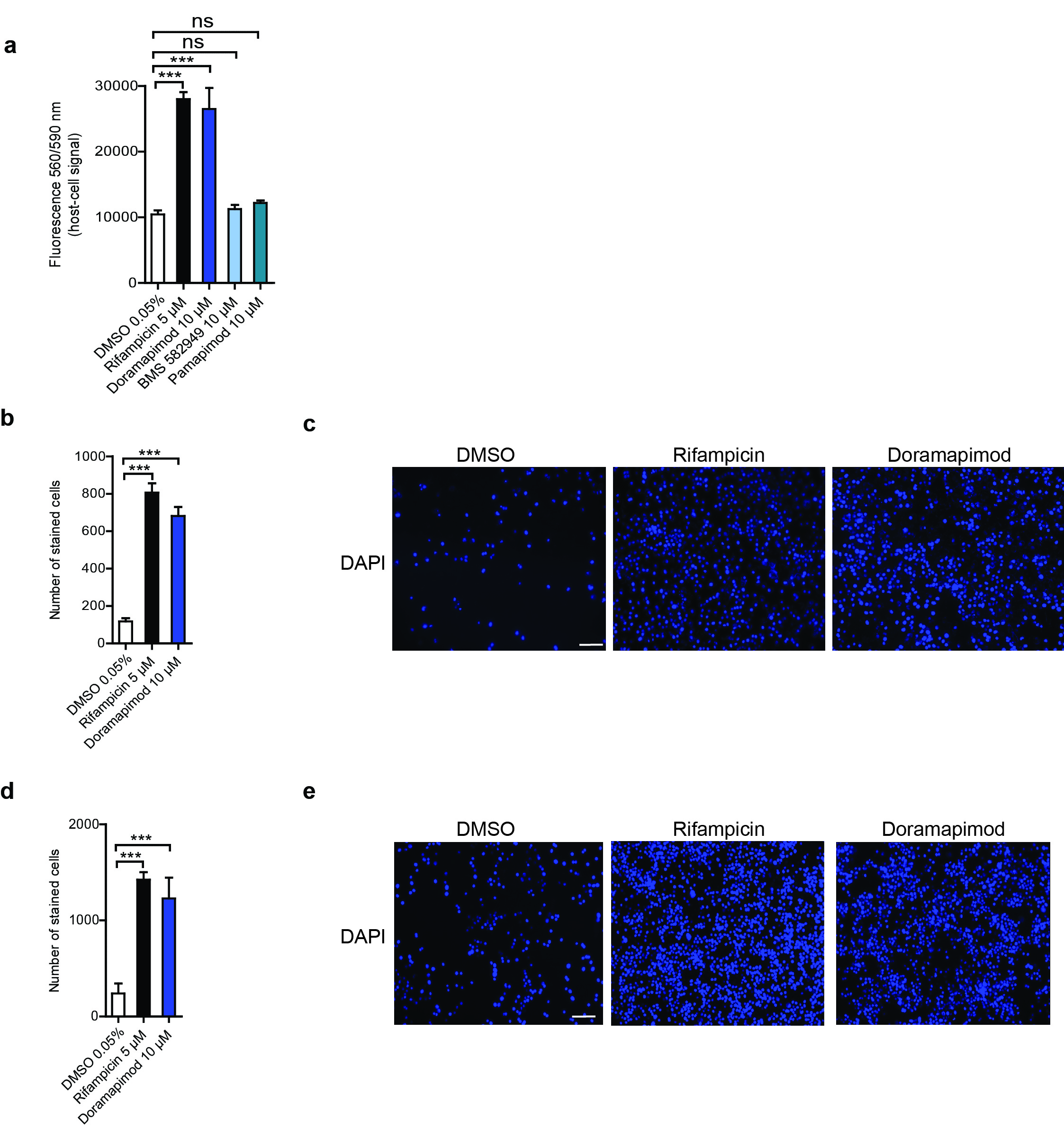
**

**Supplementary Figure 5. Several p38 MAPK inhibitors failed to abrogate mycobacterial cytotoxicity. (a)** Treatment of *Mtb*-infected (MOI 10) MRC-5 lung fibroblasts with the p38 MAPK inhibitors BMS-582949 (10 µM), pamapimod (10 µM) and doramapimod (10 µM). Viability was quantified using PrestoBlue. SB203580, another p38 MAPK inhibitor also failed to protect cells (not shown). **(b-e)** Protective effect of doramapimod in *Mtb*-infected (MOI 5) J774.2 Mφ **(b)** and BV-2 microglia **(d)** analyzed by DAPI staining with representative images shown in **c** and **e**. Results from two individual experiments are shown in **a**; data from one experiment with multiple replicates are shown in **b**; data pooled from two independent experiments are shown in **d**. Results are expressed as mean ± SEM and were analyzed using unpaired t test (ns, not significant; **, p≤ 0.01; ***, p≤ 0.001).

**Supplementary Fig. 6**

**
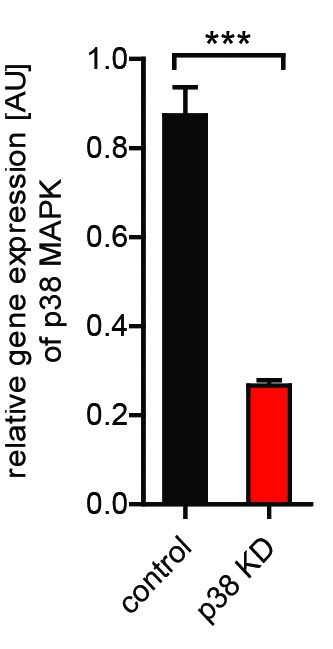
**

**Supplementary Figure 6. Knock-down of p38 MAPK in J774A.1 Mφ.** Expression of p38 MAPK in J774A.1 Mφ and J774A.1 p38 MAPK KD Mφ analyzed by qRT-PCR. Data from one experiment with multiple replicates are shown and expressed as mean ± SEM. Data were analyzed using unpaired t test (***, p≤ 0.001).

**Supplementary Fig. 7**

**
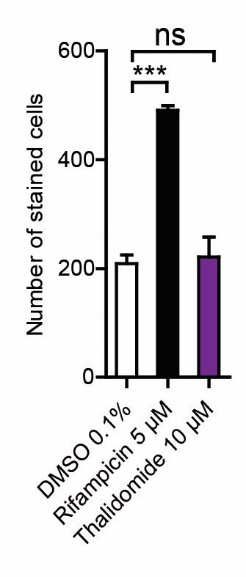
**

# Supplementary Figure 7. TNF-α inhibition has no protective effect on Mφ from TB patients. Mφ isolated from patients were infected with *Mtb* at an MOI of 1 and cell survival was quantified 48 h after infection by DAPI staining. Representative data from two experiments with multiple replicates are shown and expressed as mean ± SEM. Data were analyzed using unpaired t test (ns, not significant; ***, p≤ 0.001).

**Supplementary Fig. 8**

**
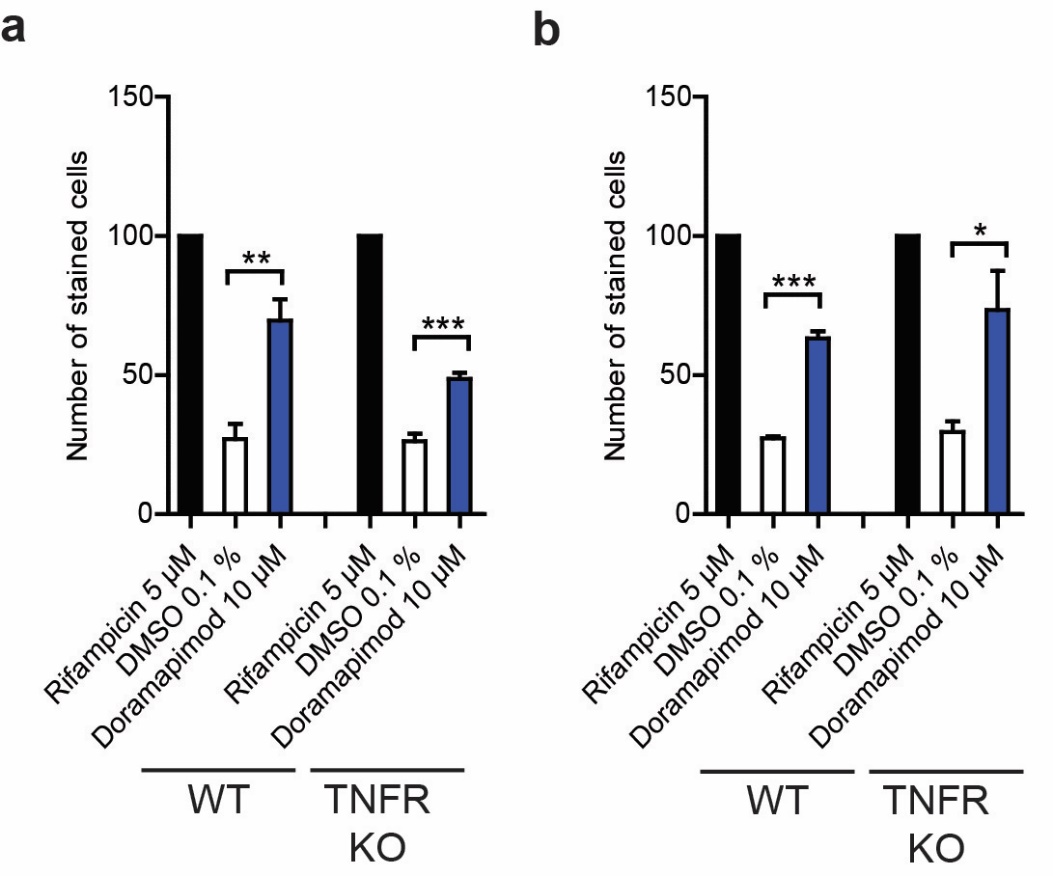
**

# Supplementary Figure 8. Doramapimod has a protective effect on wild-type and TNFR^-/-^ bone marrow-derived Mφ (BMDM). BMDM from wild-type (WT) and tumor [necrosis factor receptor](https://en.wikipedia.org/wiki/Tumor_necrosis_factor_alpha) (TNFR^-/-^) mice were infected with *Mtb* at an MOI of 3 (a) or 5 (b) and cells were quantified 48 h after infection using DAPI staining. Data from two experiment with multiple replicates are shown and expressed as mean ± SEM. Data were analyzed using unpaired t test (*, p≤ 0.05; **, p≤ 0.01; ***, p≤ 0.001).

**Supplementary Fig. 9**

**
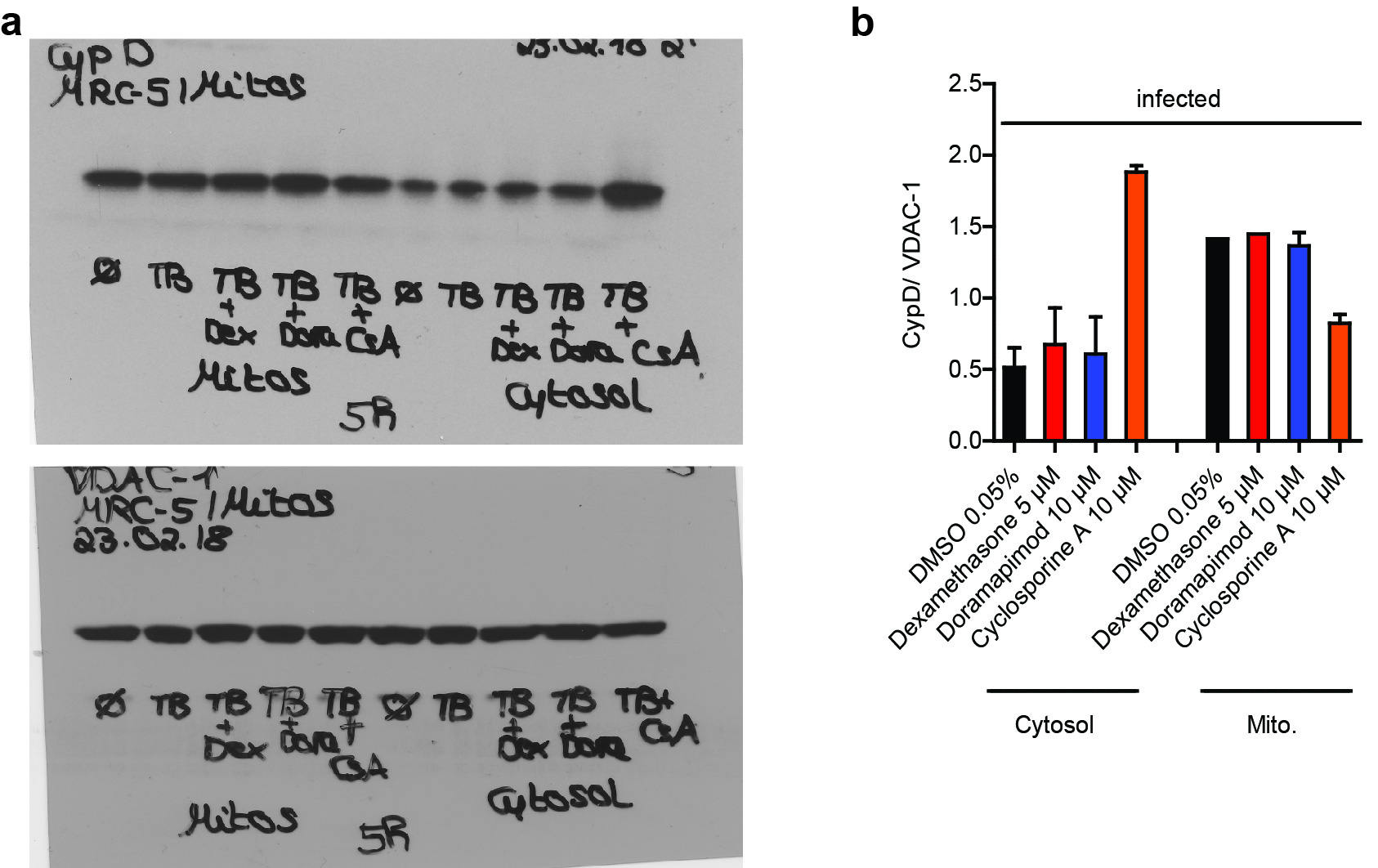
**

**Supplementary Figure 9. Dexamethasone and doramapimod have no effect on mitochondrial cyclophilin D (CypD).** Quantification of cytosolic and mitochondrial CypD following *Mtb* infection (MOI 10) in MRC-5 lung fibroblasts treated with dexamethasone (5 µM), doramapimod (10 µM) or cyclosporine A (10 µM). Lysates were obtained from mitochondria 5 h post infection. Equal amounts of protein from both the mitochondrial as well as the cytosolic fractions were subjected to Western blot analysis and the levels of CypD were measured **(a)**. Voltage-dependent anion-selective channel 1 (VDAC-1) was used as a loading control. Results are representative of two individual experiments **(b)**.

**Supplementary Fig. 10**

**
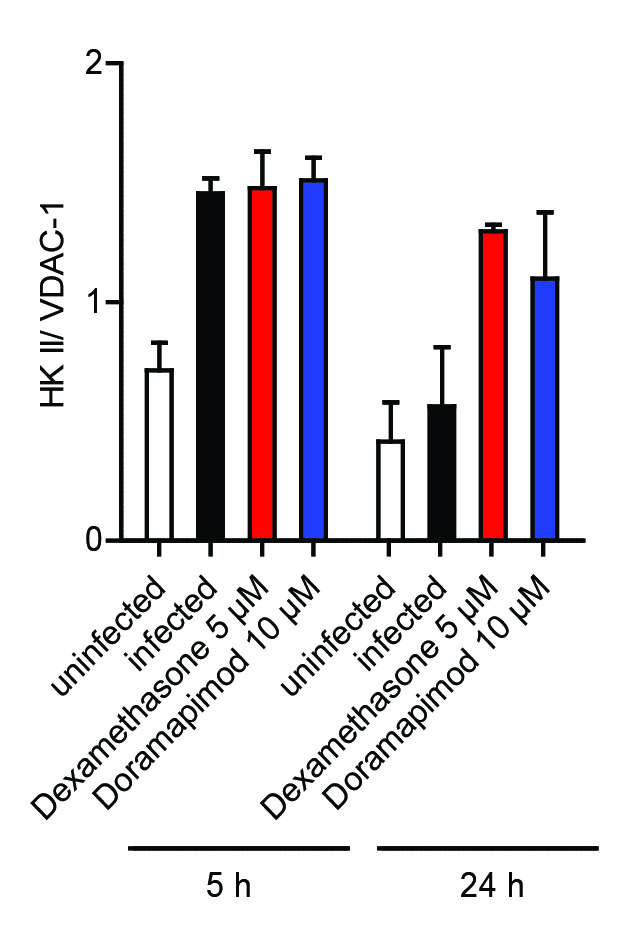
**

**Supplementary Figure 10. *Mtb* infection triggers hexokinase II (HKII) translocation to mitochondria.** Quantificiation of mitochondrial HKII in *Mtb* infected J774.2 Mφ 5 h and 24 h post infection using Western blot analysis. Infected Mφ (MOI 5) were treated with dexamethasone (5 µM) or doramapimod (10 µM) and equal amounts of protein were subjected to Western blot analysis. HKII was quantified by comparing band intensity to the loading control VDAC-1. Results are expressed as mean ± SEM of two individual experiments.

**Supplementary Fig. 11**

**
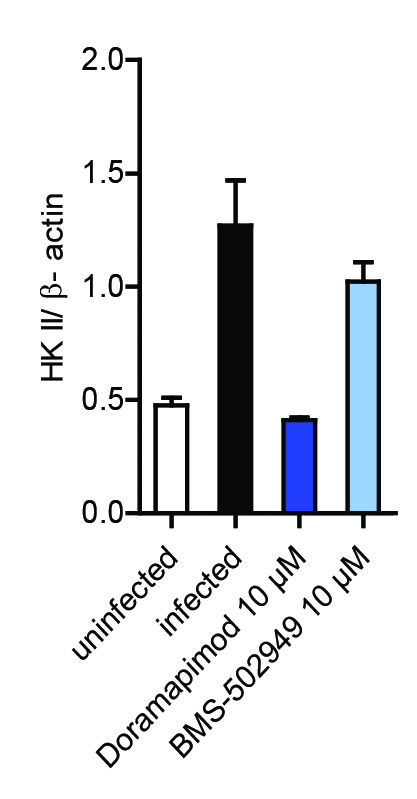
**

**Supplementary Figure 11. *Mtb* infection increases hexokinase II (HKII) expression.** Quantification of HKII expression in whole cell lysates of *Mtb* infected MRC-5 lung fibroblasts (MOI 10) 48 h post infection. Lysates were obtained from untreated cells as well as from cells treated with the p38 MAPK inhibitors doramapimod (10 µM) or BMS-582949 (10 µM). HKII was quantified by Western blot analysis using β-Actin as a loading control. Results are expressed as mean ± SEM of two individual experiments.

**Supplementary Fig.** **12**

**
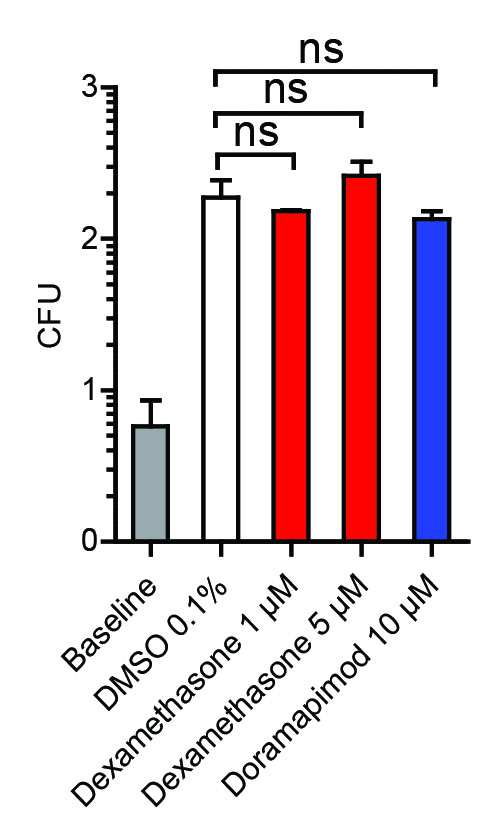
**

**Supplementary Figure 12. Dexamethasone has no effect on intracellular bacterial burden.** Determination of the colony-forming unit (CFU) of *Mtb*-infected J774.2 Mφ treated with or without dexamethasone (1 or 5 µM) or doramapimod (10 µM). Results are expressed as mean ± SEM of two individual experiments with multiple replicates. Data were analyzed using unpaired t test (ns, not significant).

**Supplementary Fig. 13**

**
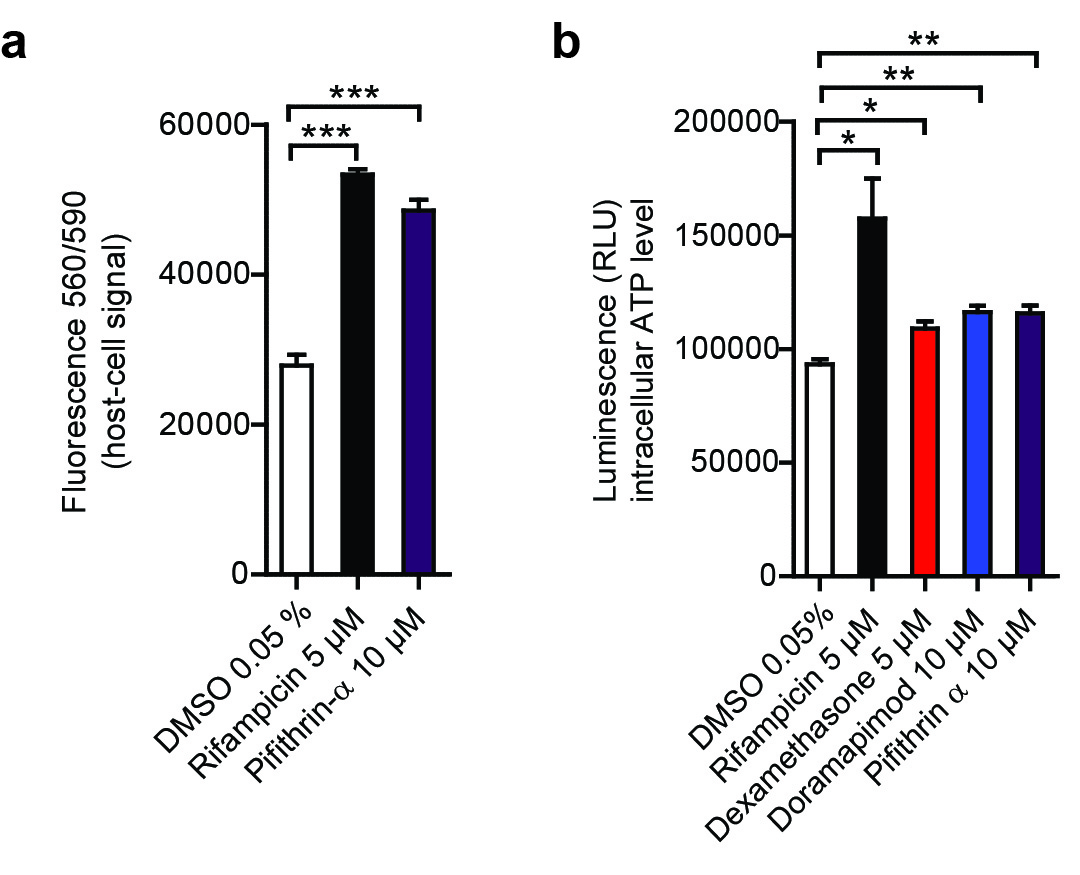
**

**Supplementary Figure 13. Chemical inhibition of p53** **is cytoprotective.** **(a)** Protective effect of the p53 inhibitor pifithrin α (10 µM) in *Mtb-*infected MRC-5 lung fibroblasts (MOI 10). Host cell viability was quantified using PrestoBlue. Data were obtained from two individual experiments with multiple replicates using the fibroblast survival assay. **(b)** Intracellular ATP levels in J774.2 Mφ following 24 h of TB infection. Cells were treated with pifithrin α (10 µM), dexamethasone (5 µM) or doramapimod (10 µM) and ATP activity was detected by luminescence. Data were obtained from one experiment with multiple replicates and are shown as mean ± SEM. Analysis was done using unpaired t test (*, p≤ 0.05; **, p≤ 0.01; ***, p≤ 0.001).

**Supplementary Table 1. Hit list of chemical library screen.** The library contained 1,280 approved drugs. Shown are hit-compounds with known antimicrobial activity and corticosteroids. The score value is relative to the control rifampicin (value of 1). Score SD represents the standard deviation from measurements of duplicate assay plates. Drug concentration was 10 µM.

| Name | Score | ScoreSD | HIT |
| --- | --- | --- | --- |
| Ethambutol dihydrochloride | 1,23 | 0,034 | YES |
| Rifabutin | 1,19 | 0,045 | YES |
| Linezolid | 1,19 | 0,031 | YES |
| Rifaximin | 1,04 | 0,001 | YES |
| Rifapentine | 1,02 | 0,090 | YES |
| Isoconazole | 0,93 | 0,102 | YES |
| Prothionamide | 0,92 | 0,052 | YES |
| Isoniazid | 0,92 | 0,020 | YES |
| Rifampicin | 0,91 | 0,012 | YES |
| Ethionamide | 0,88 | 0,038 | YES |
| Amikacin hydrate | 0,82 | 0,005 | YES |
| Dexamethasone acetate | 0,81 | 0,136 | YES |
| Prednisone | 0,75 | 0,066 | YES |
| Fluorometholone | 0,75 | 0,067 | YES |
| Oxytetracycline dihydrate | 0,74 | 0,066 | YES |
| Cyclosporin A | 0,74 | 0,012 | YES |
| Fludrocortisone acetate | 0,72 | 0,026 | YES |
| Miconazole | 0,71 | 0,008 | YES |
| Moxifloxacin | 0,70 | 0,020 | YES |
| Clobetasol propionate | 0,69 | 0,041 | YES |
| Merbromin | 0,69 | 0,083 | YES |
| Fusidic acid sodium salt | 0,66 | 0,104 | YES |
| Clarithromycin | 0,63 | 0,108 | YES |
| Minocycline hydrochloride | 0,61 | 0,039 | YES |
| Tioconazole | 0,60 | 0,132 | YES |
| Gatifloxacin | 0,59 | 0,042 | YES |
| Roxithromycin | 0,59 | 0,002 | YES |
| Beclomethasone dipropionate | 0,58 | 0,158 | YES |
| Methylprednisolone, 6-alpha | 0,57 | 0,042 | YES |
| Ivermectin | 0,57 | 0,019 | YES |
| Chlorhexidine | 0,56 | 0,113 | YES |
| Meclocycline sulfosalicylate | 0,56 | 0,034 | YES |
| Demeclocycline hydrochloride | 0,54 | 0,028 | YES |
| Alclometasone dipropionate | 0,53 | 0,140 | YES |
| Fluocinonide | 0,52 | 0,087 | YES |
| 4-aminosalicylic acid | 0,50 | 0,022 | YES |
| Tylosin | 0,48 | 0,068 | YES |
| Methacycline hydrochloride | 0,48 | 0,012 | YES |
| Megestrol acetate | 0,48 | 0,003 | YES |
| Doxycycline hydrochloride | 0,47 | 0,044 | YES |
| Nifurtimox | 0,46 | 0,035 | YES |
