# Supplementary material for: Corticosteroids inhibit Mycobacterium tuberculosis-induced necrotic host cell death by abrogating mitochondrial membrane permeability transition
Source: Nat Commun. 2019 Feb 8;10:688. doi: 10.1038/s41467-019-08405-9 (PMC6368550; doi:10.1038/s41467-019-08405-9)
Supplement: Supplementary file 2 — Reporting Summary [file 41467_2019_8405_MOESM2_ESM.pdf]

natureresearch

Corresponding author(s): *Double-blind peer review submissions: write DBPR and your manuscript number here instead of author names.*

Reporting Summary

Nature Research wishes to improve the reproducibility of the work that we publish. This form provides structure for consistency and transparency in reporting. For further information on Nature Research policies, see Authors & Referees and the Editorial Policy Checklist.

Statistical parameters

When statistical analyses are reported, confirm that the following items are present in the relevant location (e.g. figure legend, table legend, main text, or Methods section).

n/a	Confirmed	
☐	☒	The exact sample size (*n*) for each experimental group/condition, given as a discrete number and unit of measurement
☐	☒	An indication of whether measurements were taken from distinct samples or whether the same sample was measured repeatedly
☐	☒	The statistical test(s) used AND whether they are one- or two-sided<br>*Only common tests should be described solely by name; describe more complex techniques in the Methods section.*
☒	☐	A description of all covariates tested
☒	☐	A description of any assumptions or corrections, such as tests of normality and adjustment for multiple comparisons
☐	☒	A full description of the statistics including central tendency (e.g. means) or other basic estimates (e.g. regression coefficient) AND variation (e.g. standard deviation) or associated estimates of uncertainty (e.g. confidence intervals)
☒	☐	For null hypothesis testing, the test statistic (e.g. *F*, *t*, *r*) with confidence intervals, effect sizes, degrees of freedom and *P* value noted<br>*Give P values as exact values whenever suitable.*
☒	☐	For Bayesian analysis, information on the choice of priors and Markov chain Monte Carlo settings
☒	☐	For hierarchical and complex designs, identification of the appropriate level for tests and full reporting of outcomes
☒	☐	Estimates of effect sizes (e.g. Cohen's *d*, Pearson's *r*), indicating how they were calculated
☐	☒	Clearly defined error bars<br>*State explicitly what error bars represent (e.g. SD, SE, CI)*

Our web collection on statistics for biologists may be useful.

Software and code

Policy information about availability of computer code

Data collection	Excel. Graphpad prism
Data analysis	Graphpad prism

For manuscripts utilizing custom algorithms or software that are central to the research but not yet described in published literature, software must be made available to editors/reviewers upon request. We strongly encourage code deposition in a community repository (e.g. GitHub). See the Nature Research guidelines for submitting code & software for further information.

Data

Policy information about availability of data

All manuscripts must include a data availability statement. This statement should provide the following information, where applicable:

- Accession codes, unique identifiers, or web links for publicly available datasets
- A list of figures that have associated raw data
- A description of any restrictions on data availability

does not apply

Field-specific reporting

Please select the best fit for your research. If you are not sure, read the appropriate sections before making your selection.

☒ Life sciences ☐ Behavioural & social sciences ☐ Ecological, evolutionary & environmental sciences

For a reference copy of the document with all sections, see nature.com/authors/policies/ReportingSummary-flat.pdf

Life sciences study design

All studies must disclose on these points even when the disclosure is negative.

Sample size	See methods section last paragraph "statistical analysis and general methods"
Data exclusions	No samples or animals were excluded
Replication	Experiments were replicated at least twice. Results were consistent.
Randomization	Laboratory animals were randomly chosen and sepparated in cages before treatment. Treatment was performed by a person not involved in downstream experiments using animal material.
Blinding	Blinding was not performed.

Reporting for specific materials, systems and methods

Materials & experimental systems

n/a	Involved in the study	
☒	☐	Unique biological materials
☐	☒	Antibodies
☐	☒	Eukaryotic cell lines
☒	☐	Palaeontology
☐	☒	Animals and other organisms
☐	☒	Human research participants

Methods

n/a	Involved in the study	
☒	☐	ChIP-seq
☒	☐	Flow cytometry
☒	☐	MRI-based neuroimaging

Antibodies

Antibodies used	Most antibodies were purches from Cellsignal. Information was provided in the methods section.
Validation	See information provided by cellsignal

Eukaryotic cell lines

Policy information about cell lines

Cell line source(s)	See methods section.
Authentication	Is peformed by provider and by morphology check using light microscopy
Mycoplasma contamination	Contamination is checked on a regular basis using pcr techniques
Commonly misidentified lines (See ICLAC register)	does not apply

Animals and other organisms

Policy information about studies involving animals; ARRIVE guidelines recommended for reporting animal research

Laboratory animals	Female wild type C57BL/6 mice, age eight to twelve weeks

Wild animals	does not apply
Field-collected samples	does not apply

Human research participants

Policy information about studies involving human research participants

Population characteristics	Patients with confirmed active tuberculosis were chosen.
Recruitment	Patients were selected after confirmation of active tuberculosis by the in house microbiology department.
